# Iron homeostasis proteins Grx4 and Fra2 control activity of the *Schizosaccharomyces pombe* iron repressor Fep1 by facilitating [2Fe-2S] cluster removal

**DOI:** 10.1016/j.jbc.2023.105419

**Published:** 2023-11-03

**Authors:** Debolina Hati, Ariane Brault, Malini Gupta, Kylie Fletcher, Jean-François Jacques, Simon Labbé, Caryn E. Outten

**Affiliations:** 1 Department of Chemistry and Biochemistry, University of South Carolina, Columbia, South Carolina, USA; 2 Département de Biochimie et de Génomique Fonctionnelle, Faculté de médecine et des sciences de la santé, Université de Sherbrooke, Sherbrooke, Quebec, Canada

**Keywords:** iron metabolism, iron-sulfur protein, GATA-type transcription factor, metal homeostasis, protein-protein interaction, circular dichroism, monothiol glutaredoxin, BolA-like protein, fission yeast

## Abstract

The Bol2 homolog Fra2 and monothiol glutaredoxin Grx4 together play essential roles in regulating iron homeostasis in *Schizosaccharomyces pombe. In vivo* studies indicate that Grx4 and Fra2 act as coinhibitory partners that inactivate the transcriptional repressor Fep1 in response to iron deficiency. In *Saccharomyces cerevisiae,* Bol2 is known to form a [2Fe-2S]-bridged heterodimer with the monothiol Grxs Grx3 and Grx4, with the cluster ligands provided by conserved residues in Grx3/4 and Bol2 as well as GSH. In this study, we characterized this analogous [2Fe-2S]-bridged Grx4-Fra2 complex in *S. pombe* by identifying the specific residues in Fra2 that act as ligands for the Fe-S cluster and are required to regulate Fep1 activity. We present spectroscopic and biochemical evidence confirming the formation of a [2Fe-2S]-bridged Grx4-Fra2 heterodimer with His66 and Cys29 from Fra2 serving as Fe-S cluster ligands in *S. pombe. In vivo* transcription and growth assays confirm that both His66 and Cys29 are required to fully mediate the response of Fep1 to low iron conditions. Furthermore, we analyzed the interaction between Fep1 and Grx4-Fra2 using CD spectroscopy to monitor changes in Fe-S cluster coordination chemistry. These experiments demonstrate unidirectional [2Fe-2S] cluster transfer from Fep1 to Grx4-Fra2 in the presence of GSH, revealing the Fe-S cluster dependent mechanism of Fep1 inactivation mediated by Grx4 and Fra2 in response to iron deficiency.

The trace metal iron is essential for the survival of almost all living organisms since it serves a cofactor for proteins involved in fundamental biochemical functions such as respiration, nitrogen fixation, and DNA and amino acid biosynthesis, to name a few. Iron homeostasis in the nonpathogenic fungi *Saccharomyces cerevisiae* and *Schizosaccharomyces pombe* has been extensively studied to understand how iron is regulated under different environmental conditions (1, 2, 3). The iron regulation pathways in these two yeasts are mechanistically distinct, but they both rely on the use of iron-sulfur (Fe-S) clusters as signals to balance iron acquisition, storage, and utilization in the cell. In the budding yeast *S. cerevisiae*, an inhibitory Fe-S cluster is delivered from the [2Fe-2S]-bridged Grx3/4-Bol2 heterodimer to transcriptional activators Aft1 and Aft2 during iron sufficiency to alter their oligomeric state and trigger their dissociation from DNA, which in turn deactivates the expression of iron uptake genes (4, 5, 6, 7). In the fission yeast *S. pombe*, there are no transcription factors homologous to Aft1/Aft2, but the cytosolic glutaredoxin (Grx) Grx4 and BolA-like protein Fra2 play analogous roles to their *S. cerevisiae* orthologs by controlling the activity of the transcriptional repressors Php4 and Fep1 (1, 2, 8). Under iron deplete conditions, Php4 is found in the nucleus bound to the CCAAT-binding complex (Php2/3/5) to repress iron utilization genes. When iron levels increase, a [2Fe-2S] cluster-dependent interaction between Grx4 and Php4 facilitates Php4’s dissociation from Php2/3/5 and export to the cytosol, thereby derepressing iron utilization genes (9, 10, 11). Whereas in iron replete conditions, the GATA-type regulator Fep1 interacts with the promoters of its target genes downregulating iron uptake. Conversely, in iron deplete conditions, Grx4 and Fra2 together promote Fep1’s dissociation from its DNA targets, leading to the derepression of iron acquisition genes (12, 13, 14, 15).

Previous studies have provided some insight into the posttranslational, iron-dependent mechanism by which Grx4 and Fra2 inhibit Fep1 activity. *S. pombe* Grx4 is a typical multidomain, class II Grx, also called a monothiol Grx, that forms a [2Fe-2S]-cluster bridged homodimer *via* its Grx domain (11, 12). The Fe-S cluster is ligated by two GSH molecules as well as Cys172 of the conserved Cys-Gly-Phe-Ser (CGFS) active site motif from each Grx4 monomer. This conserved Cys172 residue is essential for the physical interaction between Grx4 and Fep1 that drives inhibition of Fep1 in response to low-iron conditions (12, 14). *S. pombe* Fra2, a member of the BolA2 protein subfamily (8), associates with Fep1 in an iron-independent manner in the nucleus and forms a complex with both Grx4 and Fep1 *in vivo* (13). However, the mechanism by which Fra2 contributes to the inactivation of Fep1 is not clear. Furthermore, the specific iron cofactor bound by *S. pombe* Fep1 requires further study, since mononuclear iron, [2Fe-2S], [3Fe-4S], or [4Fe-4S] clusters have all been proposed (12, 16, 17).

In *S. cerevisiae*, His103 and Cys66 residues in Fra2 are important for the [2Fe-2S]-bridged association between Fra2 and Grx3 (4). Since these two amino acids are conserved in *S. pombe* Fra2 (corresponding to Cys29 and His66), we tested their role in the metal-binding interaction between Fra2 and Grx4 *in vitro*, and regulation of Fep1 activity *in vivo*. Our results confirm that these two residues are required for stable complex formation between Fra2 and Grx4 and derepression of Fep1-regulated genes in response to low iron. To address the specific type of Fe cofactors bound by Fep1 that mediates its interactions with Grx4-Fra2, we characterized the metal-binding properties of as-purified Fep1 and evaluated its interaction with Grx4-Fra2 using CD spectroscopy, which is exquisitely sensitive to the coordination chemistry of Fe-S cluster-binding proteins. These experiments demonstrate that Grx4 and Fra2 promote transfer of the [2Fe-2S] cluster from Fep1 to Grx4-Fra2. Taken together, these studies provide new insight into the mechanism by which Fra2 and Grx4 work together to regulate the repressor activity of Fep1 in response to iron bioavailability.

## Results

### *S. pombe* Fra2 binds to the [2Fe-2S] cluster ligated by Grx4

**Figure 1:**
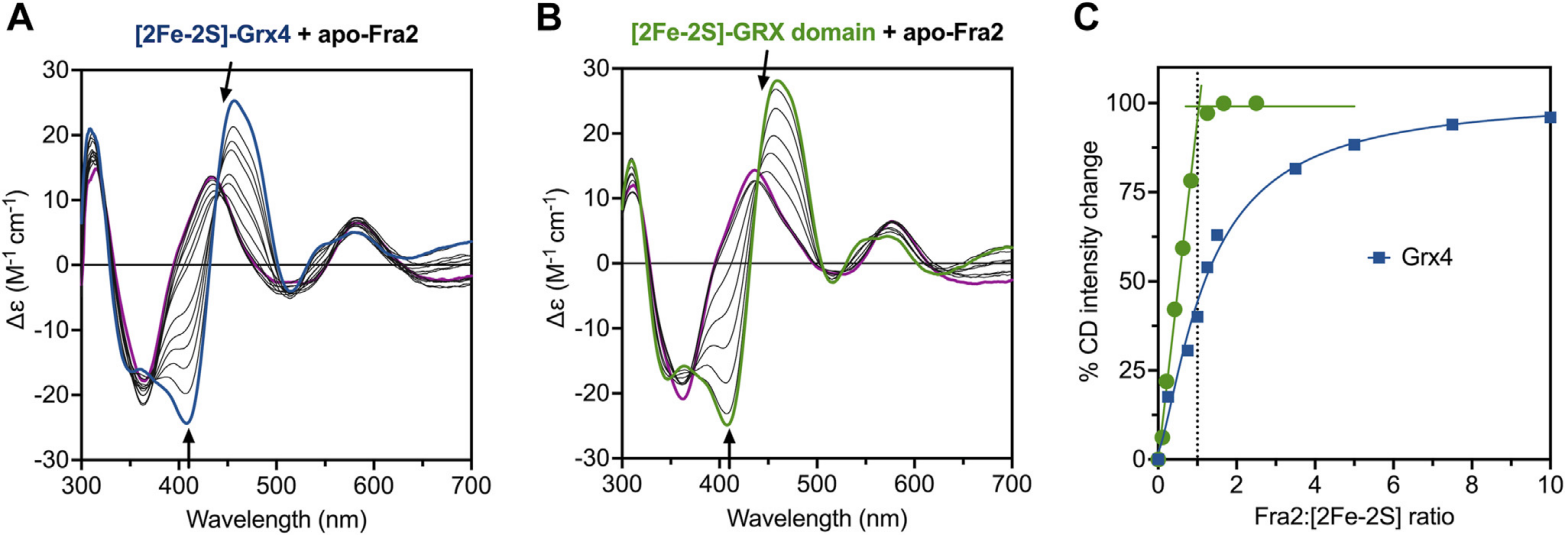
**CD-monitored titration studies of [2Fe-2S]-Grx4 and [2Fe-2S]-GRX domain with WT apo-Fra2.***A*, [2Fe-2S]-Grx4 (*thick blue line*) was titrated with increasing concentrations of Fra2 (*thin black lines*). A 25-fold excess Fra2 is shown as a *thick purple line*. *B*, [2Fe-2S]-GRX domain (*thick green line*) was titrated with a 0.125- to 3-fold excess of Fra2 (*thin black lines*). A 3-fold excess Fra2 is shown as a *thick purple line*. The arrows at selected wavelengths indicate the direction of intensity change with increasing Fra2 concentration. Δε values are based on the [2Fe-2S] cluster concentration (50 μM for [2Fe-2S]-Grx4 and 60 μM for [2Fe-2S]-GRX domain). *C*, the percent CD intensity change (between 408 and 458 nm) for spectra in A and B was plotted as a function of the Fra2:[2Fe-2S] ratio.

Previous reports have demonstrated that *S. pombe* Grx4 harbors a [2Fe-2S]^2+^ cluster with spectroscopic features similar to other members of the CGFS Grx family (11, 12). Furthermore, cysteine desulfurase mediated Fe-S reconstitution of Grx4 in the presence of Fra2 yielded a UV-visible absorption spectrum that suggested Fe-S cluster binding (12). However, the specific cluster coordination and stoichiometry of this interaction was not explored. To address this issue, we titrated apo-Fra2 into anaerobically purified [2Fe-2S]-Grx4 and monitored changes in the metal coordination environment using CD spectroscopy. The CD spectra of CGFS Grx homodimers and Grx-BolA heterocomplexes are significantly different (4, 6, 18, 19, 20, 21, 22), which provides a convenient handle to monitor the changes in cluster ligation upon Fra2 binding. Addition of Fra2 resulted in a shift in the prominent positive and negative CD peaks at 450 and 408 nm to 434 nm and 364 nm, respectively, indicating changes in the cluster ligation and/or chirality (Fig. 1*A*). These spectral changes occurred rapidly (<10 min) and resembled previous CD spectra that we reported for titration of *S. cerevisiae* [2Fe-2S]-Grx3 with *S. cerevisiae* Bol2 (previously named Fra2) to form the [2Fe-2S]-Grx3-Bol2 heterodimer (4). We evaluated the energetics of this Fe-S cluster exchange reaction by measuring the equilibrium constant (*K*_ex_) for the CD-monitored titration (Table 1). The reaction is favorable under these conditions (*K*_ex_ = 28.1 ± 2.5), However, more than 10 mol equivalents of Fra2 (>500 μM) are required for maximal binding to Grx4 (Fig. 1*C*), which contrasts with the orthologous *S. cerevisiae* proteins that clearly demonstrated 1:1 stoichiometry under similar conditions (4).

**Table 1:** *K*_ex_ measurements for Fe-S cluster exchange reactions shown in Figures 1, 3 and 6

Fe-S cluster donor	Apo acceptor	*K*_ex_
[2Fe-2S]-Grx4	WT Fra2	28.1 ± 2.5
[2Fe-2S]-GRX domain	WT Fra2	440 ± 160
[2Fe-2S]-GRX domain	C29A Fra2	6.2 ± 1.3
[2Fe-2S]-GRX domain	H66A Fra2	1.1 ± 0.2
[2Fe-2S]-GRX domain	C29A/H66A Fra2	No exchange
[2Fe-2S],Zn-Fep1-DBD	Grx4	5.3 ± 1.2
[2Fe-2S],Zn-Fep1-DBD	GRX domain	1.4 ± 0.2
[2Fe-2S],Zn-Fep1-DBD	Grx4-Fra2	36.1 ± 9.3
[2Fe-2S],Zn-Fep1-DBD	GRX domain-Fra2	3.9 ± 0.6
[2Fe-2S],Zn-Fep1-DBD	Fra2	No exchange
[2Fe-2S]-Grx4	Zn-Fep1-DBD	No exchange
[2Fe-2S]-GRX domain	Zn-Fep1-DBD	No exchange
[2Fe-2S]-Grx4-Fra2	Zn-Fep1-DBD	No exchange
[2Fe-2S]-GRX domain-Fra2	Zn-Fep1-DBD	No exchange

Since we previously reported that full-length Grx4 tends to aggregate (11), we reasoned that this aggregation may interfere in Fra2 binding. To address this issue, we expressed and purified the GRX domain alone (amino acids 143–244). UV-visible absorption and CD spectroscopic analysis of this truncated form indicated binding of a [2Fe-2S]^2+^ cluster that closely matched the full-length protein (Fig. S2). These findings parallel the previously published results for multidomain CGFS Grxs, demonstrating that the C-terminal GRX domain harbors the [2Fe-2S] cluster binding site (8, 23, 24). Titration of the [2Fe-2S]-GRX domain with apo-Fra2 generated similar CD spectra changes as observed for full-length [2Fe-2S]-Grx4 (Fig. 1*B*); however, the CD signal intensity change reached saturation after only 1 mol equivalent of Fra2 was added per [2Fe-2S] cluster (Fig. 1*C*), indicating 1:1 stoichiometry for this complex. Accordingly, the *K*_ex_ for this cluster exchange reaction is ∼16-fold higher than for the equivalent reaction with full-length Grx4 (Table 1). This difference in interaction thermodynamics may be due to the aggregation of full-length Grx4. Alternatively, it is possible that the N-terminal thioredoxin (TRX) domain of full-length Grx4 may weaken the interaction between the C-terminal GRX domain and Fra2.

### Fra2 and Grx4 form a heterodimeric complex bridged by a [2Fe-2S] cluster

**Figure 2:**
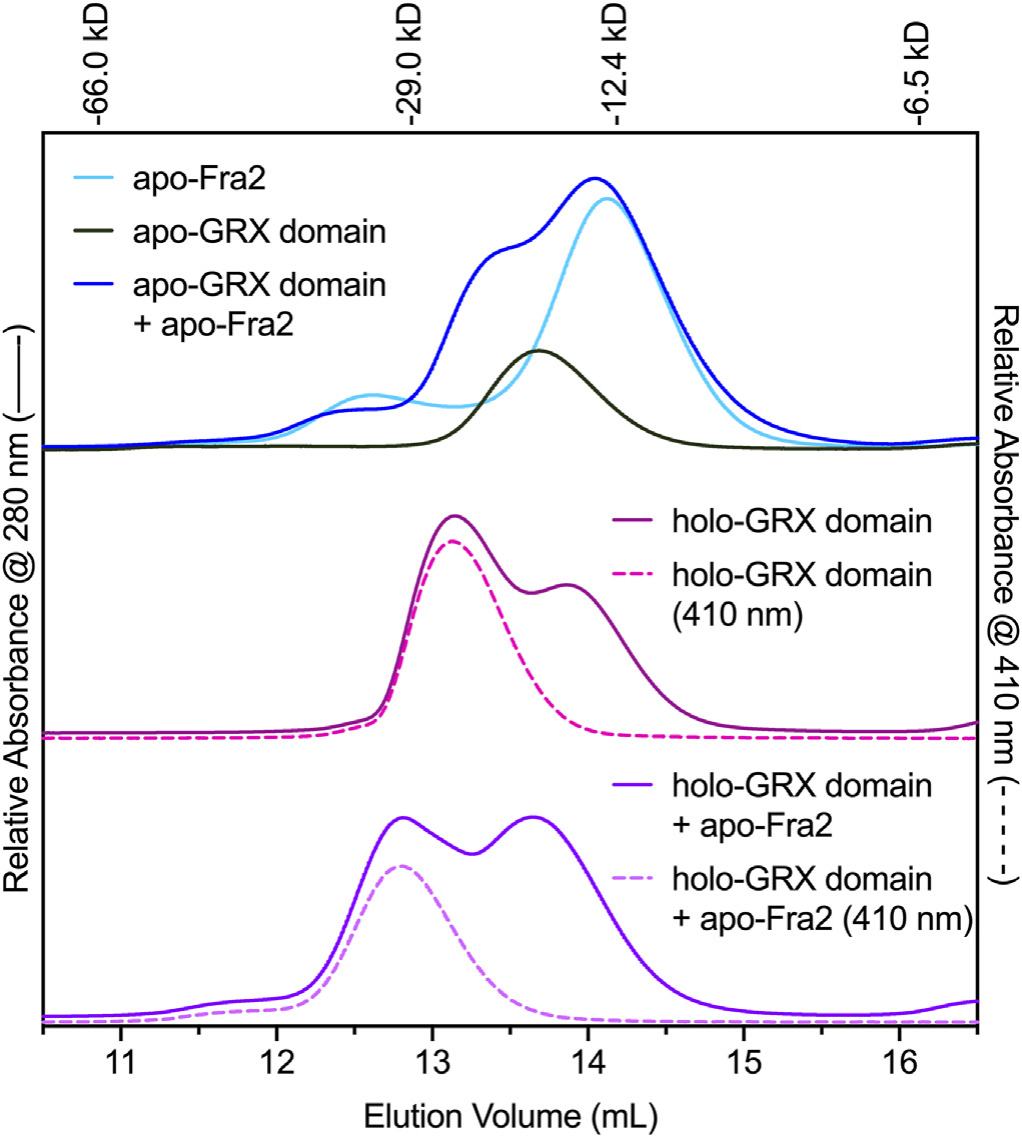
**Size-exclusion chromatography analysis of GRX domain-Fra2 interactions**. *Top*, chromatograms of apo-Fra2 (*light blue line*), apo-GRX domain (*black line*), and 1:1 mixture of apo-GRX domain + apo-Fra2 (*dark blue line*). *Middle*, [2Fe-2S] GRX domain (*pink lines*). *Bottom*, 1:1 mixture of [2Fe-2S]-GRX domain + apo-Fra2 (*purple lines*). All *solid line* chromatograms are obtained from absorbance at 280 nm, while *dashed lines* depict absorbance at 410 nm indicating [2Fe-2S] cluster binding (0.1–0.4 mg protein loaded). The elution positions and sizes of the molecular mass standards are shown above the chromatograms.

To confirm the stoichiometry of the binding interaction between [2Fe-2S]-GRX domain and Fra2, we subjected these samples to mass spectrometry (MS) and gel-filtration chromatography (Fig. 2 and Table 2). The MS results confirmed the expected molecular mass of 6xHis-Fra2 with the N-terminal Met removed. Interestingly, the MS results were different for apo-GRX domain *versus* holo-GRX domain. The molecular mass of holo-GRX domain matched the theoretical mass of 6xHis-GRX domain with the N-terminal Met removed. However, the mass of apo-GRX domain was 306 Da larger, likely corresponding to glutathionylation of the single Cys residue in the CGFS active site (Table 2). The only difference between the preparation of these samples was anaerobic purification of the holo protein (to preserve the O_2_-sensitive Fe-S cluster) *versus* aerobic purification of the apo protein. Since GSH was included in the purification buffers, we suspect that oxidized GSSG generated under aerobic conditions may have promoted glutathionylation of the Cys residue in the apo protein.

**Table 2:** Molecular mass analysis of Fra2 and GRX domain complexes

Sample	Gel filtration (Da)	Oligomeric state	Theoretical (Da)	ESI-MS^a^
apo-GRX domain	18210	monomer	12832	13138
apo-Fra2	14920	monomer	10878	10878
[2Fe-2S] GRX domain	23140 (Peak 1)	[2Fe-2S]-bridged homodimer	26452	12832
	16710 (Peak 2)	monomer	12832	12832
Apo GRX domain + apo-Fra2 (1:1)	19310 (Peak 1)	GRX monomer	12832	ND
	15470 (Peak 2)	Fra2 monomer	10878	ND
[2Fe-2S] GRX domain + apo-Fra2 (1:1)	26980 (Peak 1)	[2Fe-2S]-bridged heterodimer	24192	ND
	18540 (Peak 2)	GRX monomer	12832	ND

Peaks are numbered from left to right on the chromatograms. All masses are shown in Da.

a ND: Not determined.

In the gel-filtration chromatography experiment, apo-Fra2 eluted at an apparent molecular mass of 14.9 kDa, while apo-GRX domain eluted at 18.2 kDa (Fig. 2 and Table 2). These values are somewhat larger than the theoretical molecular masses of the monomer proteins; however, this is typically observed with CGFS Grxs and BolA proteins (6, 18, 19). We observed that the [2Fe-2S]-GRX domain alone eluted as two peaks with the major peak at 23.1 kDa, likely corresponding to the [2Fe-2S]-bridged homodimer as evidenced by the 410 nm absorbance, and the minor peak closer to the monomer mass (Fig. 2 and Table 2). Since the [2Fe-2S]-GRX domain sample only contains 30 to 40% [2Fe-2S] cluster per protein dimer, we would expect to see the apo monomer protein in this gel filtration experiment. The [2Fe-2S]-GRX domain-Fra2 sample was found to coelute with a higher apparent molecular mass than the individual proteins that suggested heterodimer formation at 1:1 stoichiometry. The apparent molecular mass of this complex (27.0 kDa) is larger than the [2Fe-2S]-GRX domain homodimer (23.1 kDa), suggesting a less compact structure. This heterodimer peak was not observed for the apo-GRX domain + apo-Fra2 mixture, indicating that [2Fe-2S] binding promotes the interaction.

### His66 and Cys29 are Fe-S ligands in the Grx4-Fra2 heterodimeric complex

**Figure 3:**
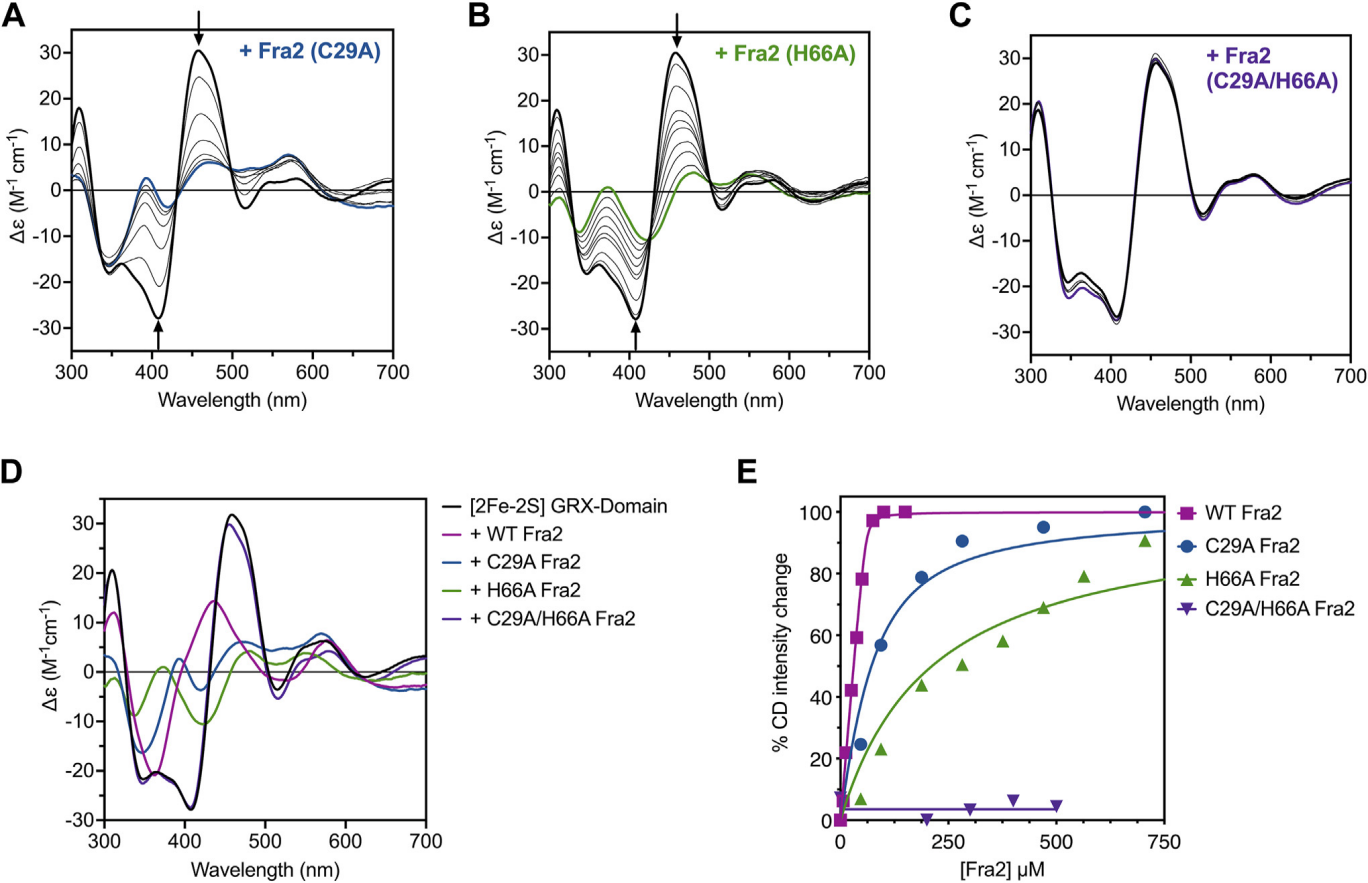
**CD-monitored titration of [2Fe-2S]-GRX domain with Fra2(C29A), Fra2(H66A), and Fra2(C29A,H66A).** [2Fe-2S]-GRX domain homodimer was titrated with 1- to 15-fold equivalents of apo Fra2(C29A) (*A*), 1- to 20-fold equivalents of apo Fra2(H66A) (*B*) or 1- to 5-fold equivalents of apo Fra2(H66A, C29A) (*C*). *D*, comparison of the CD spectra of [2Fe-2S]-GRX domain, [2Fe-2S]-GRX domain + WT Fra2, [2Fe-2S]-GRX domain + Fra2(C29A), [2Fe-2S]-GRX domain + Fra2(C66A), and [2Fe-2S]-GRX domain + Fra2(C29A/H66A). Δε values are based on the [2Fe-2S] cluster concentration (47 μM for *A* and *B*, and 100 μM for *C*). *Arrows* at selected wavelengths indicate the direction of change in peak intensity with increasing Fra2 concentrations. In *A–C*, *thick black lines* are the CD spectra of [2Fe-2S]-GRX domain alone and *blue*, *green*, and *purple lines* are the final titration mixtures. *E*, % CD intensity changes (between 408 and 458 nm) from spectra in *A–C* (Fra2 mutants) and Figure 1*B* (WT Fra2) are plotted as a function of the Fra2 concentration. The points were fit to Equation 8 to calculate the *K*_eq_ for each Fe-S exchange reaction (Table 1).

Since Cys66 and His103 of *S. cerevisiae* Bol2 were shown to be important for formation of the [2Fe-2S]-Grx3-Bol2 heterodimer in *S. cerevisiae* (4), we tested the role of the corresponding conserved amino acid residues in *S. pombe* Fra2, namely Cys29 and His66. We generated His66 to Ala (H66A) and Cys29 to Ala (C92A) mutations individually and together to assess how eliminating these putative metal-binding residues affected the Grx4:Fra2 interaction and cluster binding. We performed CD-monitored titrations of [2Fe-2S]-GRX domain homodimer with these Fra2 variants to test their interaction. As seen in Figure 3, *A* and *B*, Fra2(C29A) and Fra2(H66A) apparently bind at or near the cluster in [2Fe-2S] GRX domain as evidenced by clear changes in the CD spectra. However, the final CD spectrum of each [2Fe-2S]-GRX domain-Fra2 variant looks significantly different from the [2Fe-2S]-GRX domain-Fra2 WT complex (Fig. 3*D*), which suggests alterations in the cluster coordination environment. These findings parallel similar results reported for the *S. cerevisiae* orthologs in which single substitutions of the conserved His/Cys residues did not abolish Fe-S cluster binding but led to changes in the Fe-S cluster coordination (4). In the case of the *S. pombe* proteins, saturation of the CD signal changes required higher concentrations of Fra2 for the C29A and H66A variants than WT Fra2, corresponding to significantly lower *K*_ex_ values that suggest weaker interactions with the [2Fe-2S]-GRX domain (Table 1). Apparently, mutation of either of these residues interferes in the specific binding of apo Fra2 to [2Fe-2S]-GRX domain. We also added increasing equivalents of the Fra2(C29A,H66A) double mutant into [2Fe-2S]-GRX domain but observed no apparent Fe-S coordination changes in this case (Fig. 3*C*). These results further support assignment of both His66 and Cys29 from Fra2 as Fe-S cluster ligands in the [2Fe-2S]-Grx4-Fra2 heterodimer.

### Cells expressing fra2C29A-Myc_13_, fra2H66A-Myc_13_, and fra2C29A-H66A-Myc_13_ mutant alleles exhibit growth defects on iron-poor media

**Figure 4:**
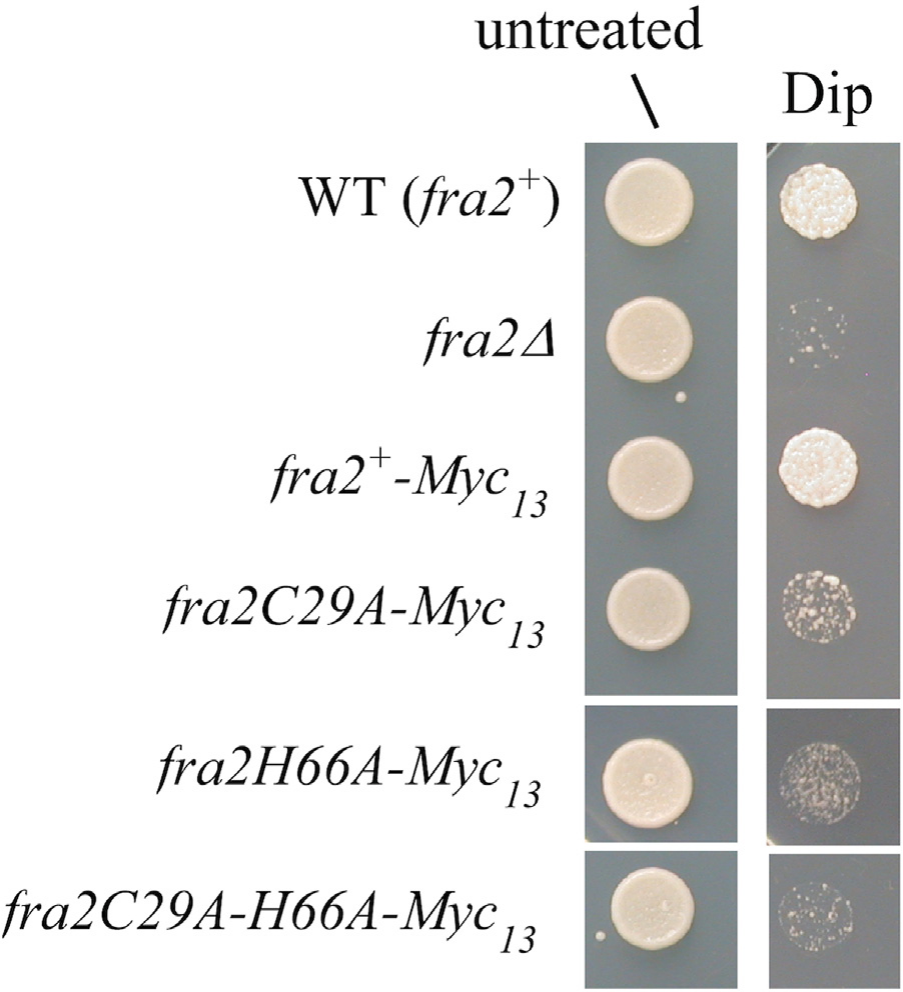
**Cys29 and His66 amino acid residues of Fra2 are required to allow cell growth under low-iron conditions**. Cells expressing *fra2*^*+*^*-Myc*_*13*_, *fra2C29A-Myc*_*13*_, *fra2H66A-Myc*_*13*_, and *fra2C29A-H66A-Myc*_*13*_ alleles were spotted onto YES medium that was left untreated or supplemented with Dip (150 μM). As controls, WT (*fra2*^*+*^) and *fra2Δ* strains were assayed under the same conditions. Once spotted on the untreated and iron-starved media, the strains were incubated for 4 days at 30 °C, and photographed.

Our previous studies showed that Fra2 was required to inhibit Fep1 function when cells undergo a transition from high to low iron (13). Consistently, the absence of Fra2 results in a constitutive repression of genes encoding proteins involved in high-affinity iron transport. Therefore, *fra2Δ* mutant cells exhibit poor growth on iron-starved medium in comparison to WT cells (13). Based on this phenotype, we further investigated whether *S. pombe* cells expressing either a *fra2C29A-Myc*_*13*_, *fra2H66A-Myc*_*13*_, or *fra2C29A-H66A-Myc*_*13*_ fusion mutant gene integrated at the chromosomal locus of *fra2*^*+*^ exhibited poor growth on low iron medium. Analysis of the steady-state mRNA and protein levels of the Myc-tagged forms confirmed their similar expression under different iron growth conditions in *S. pombe* cells (Fig. S3). However, the growth assay results consistently showed that cells expressing the C29A, H66A, and C29A-H66A variants grew poorly on medium that was supplemented with the iron chelator dipyridyl (Dip) (Fig. 4). In contrast, cells carrying an untagged or Myc_13_-tagged *fra2*^*+*^ allele grew robustly on medium containing Dip (Fig. 4). As mentioned above, the *fra2Δ* mutant used as a control strain is unable to grow on low iron medium. Together, these data suggest that the growth defect displayed by the cells expressing *fra2C29A-Myc*_*13*_, *fra2H66A-Myc*_*13*_, or *fra2C29A-H66A-Myc*_*13*_ mutant alleles under low iron is due to a lack of iron limitation-dependent inhibition of Fep1, resulting in an invariable repression of iron transport genes. Consequently, since Cys29 and His66 are both required for stable Fe-S cluster binding by the Fra2-Grx4 complex, these results indicate that Fe-S binding by Fra2 is essential for regulation of Fep1 activity.

### Expression of fra2 mutants partially or completely inhibit iron limitation-dependent induction of frp1^+^ mRNA levels

**Figure 5:**
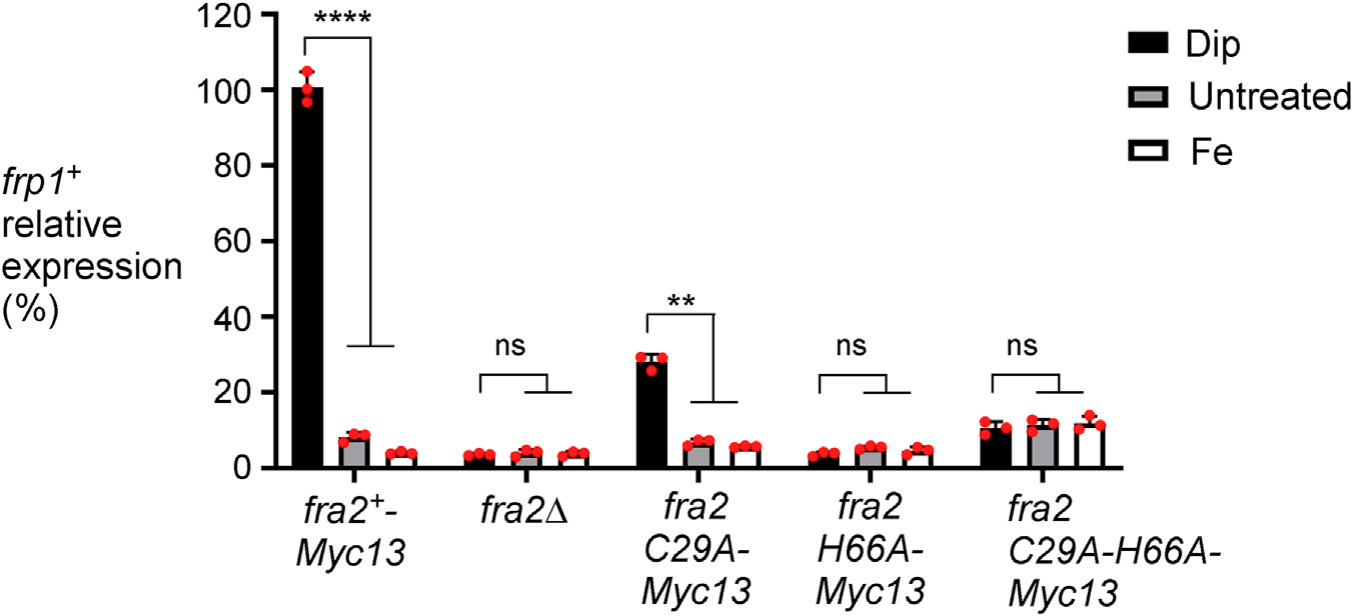
**Effects of the expression of *fra2***^***+***^***-Myc***_***13***_**, *fra2C29A-Myc***_***13***_**, *fra2H66A-Myc***_***13***_**, and *fra2C29A-H66A-Myc***_***13***_**alleles on the transcriptional response of *frp1***^***+***^**to iron starvation.** Representative expression profile of the *frp1*^*+*^ transcript in cells expressing the WT *fra2*^*+*^*-Myc*_*13*_ or its mutant derivatives that were left untreated (−) or were incubated in the presence of Dip (250 μM) or FeCl_3_ (Fe, 100 μM) for 90 min. Total RNA was prepared from culture aliquots, and steady-state mRNA levels of *frp1*^*+*^ and *act1*^*+*^ were analyzed by RT-qPCR assays. Graphic representation of quantification of three independent RT-qPCR assays. Error bars indicate the standard deviation (±SD; error bars). The *asterisks* correspond to *p* ˂ 0.01 (∗∗) and *p* < 0.0001 (∗∗∗∗) (two-way ANOVA with Tukey’s multiple comparisons test against the indicated strain grown under low-iron conditions), whereas ns stands for not significant.

Based on the finding that cells expressing *fra2C29A-Myc*_*13*_, *fra2H66A-Myc*_*13*_, and *fra2C29A-H66A-Myc*_*13*_ alleles resulted in poor growth on low iron medium, we tested whether *frp1*^*+*^, a gene known to encode a component of the iron transport machinery, was properly regulated in response to changes in iron concentrations. Using reverse transcription-quantitative PCR assays, we monitored *frp1*^*+*^ transcript levels in WT (*fra2*^*+*^*-Myc*_*13*_) and *fra2C29A-Myc*_*13*_, *fra2H66A-Myc*_*13*_, and *fra2C29A-H66A-Myc*_*13*_ mutant cells grown in either the absence or presence of Dip or iron. The gene expression levels of *frp1*^*+*^ in *fra2Δ*, *fra2H66A-Myc*_*13*_, and *fra2C29A-H66A-Myc*_*13*_ mutant alleles were repressed under basal, iron-depleted, and iron-replete conditions (Fig. 5). In the case of these mutant strains, there was a lack of induction of *frp1*^*+*^ mRNA levels in response to iron starvation compared to the levels of *frp1*^*+*^ observed in the WT strain under the same conditions. In the case of cells expressing the *fra2C29A-Myc*_*13*_ allele, *frp1*^*+*^ mRNA levels displayed a low, but significant, increase (4.1-fold) of expression in the presence of Dip compared with basal levels of expression observed in untreated cells (Fig. 5). In a WT strain expressing *fra2*^*+*^*-Myc*_*13*_ (used as a control), *frp1*^*+*^ transcript levels were induced 12.2-fold in the presence of Dip compared to levels under basal conditions (Fig. 5). Taken together, analysis of a strain lacking *fra2*^*+*^ (*fra2Δ*) or expressing a *fra2C29A-Myc*_*13*_, *fra2H66A-Myc*_*13*_, or *fra2C29A-H66A-Myc*_*13*_ mutant allele strengthened the conclusion that Fe-S binding by Fra2 is required for inhibition of Fep1 activity in iron-starved cells.

### Grx4 and Fra2 facilitate [2Fe-2S] cluster transfer from holo-Fep1

We next characterized the Fe-S cluster-dependent interactions between Grx4, Fra2, and Fep1 to probe the molecular mechanism of Grx4-Fra2-mediated regulation of Fep1. We recombinantly expressed and purified full-length Fep1 and the N-terminal, DNA-binding domain (DBD) of Fep1 (Fep1-DBD) under anaerobic conditions. We noted that full-length Fep1 is susceptible to degradation as previously reported (12, 17); therefore, we used the more stable, N-terminal Fep1-DBD for our studies (Fig. S1). Fep1-DBD harbors the two zinc finger motifs as well as an intervening Cys-rich sequence that binds the Fe cofactor (25, 26). Both Fep1 and Fep1-DBD exhibit UV-visible absorption spectra that are similar to previous reports (12, 17), with peaks at 323 and 418 nm, a shoulder at 450 nm, and an unresolved broad brand at 550 nm (Fig. S4). These features are usually indicative of an [2Fe-2S]^2+^ cluster (18, 27). However, the CD spectra of Fep1 and Fep1-DBD are relatively weaker than most other [2Fe-2S]^2+^-binding proteins (Fig. S4), which might reflect a distortion in the typical [2Fe-2S] binding coordination environment (28). Metal and acid-labile sulfide analysis of anaerobically purified Fep1-DBD from three independent samples indicated 0.76 ± 0.12 Zn, 0.69 ± 0.08 Fe, and 0.85 ± 0.10 S^2-^ bound per monomer. The measured Fe:S ratio (0.8:1) further supports Fe-S cluster binding by *S. pombe* Fep1 (17) rather than mononuclear Fe as previously reported (12).

**Figure 6:**
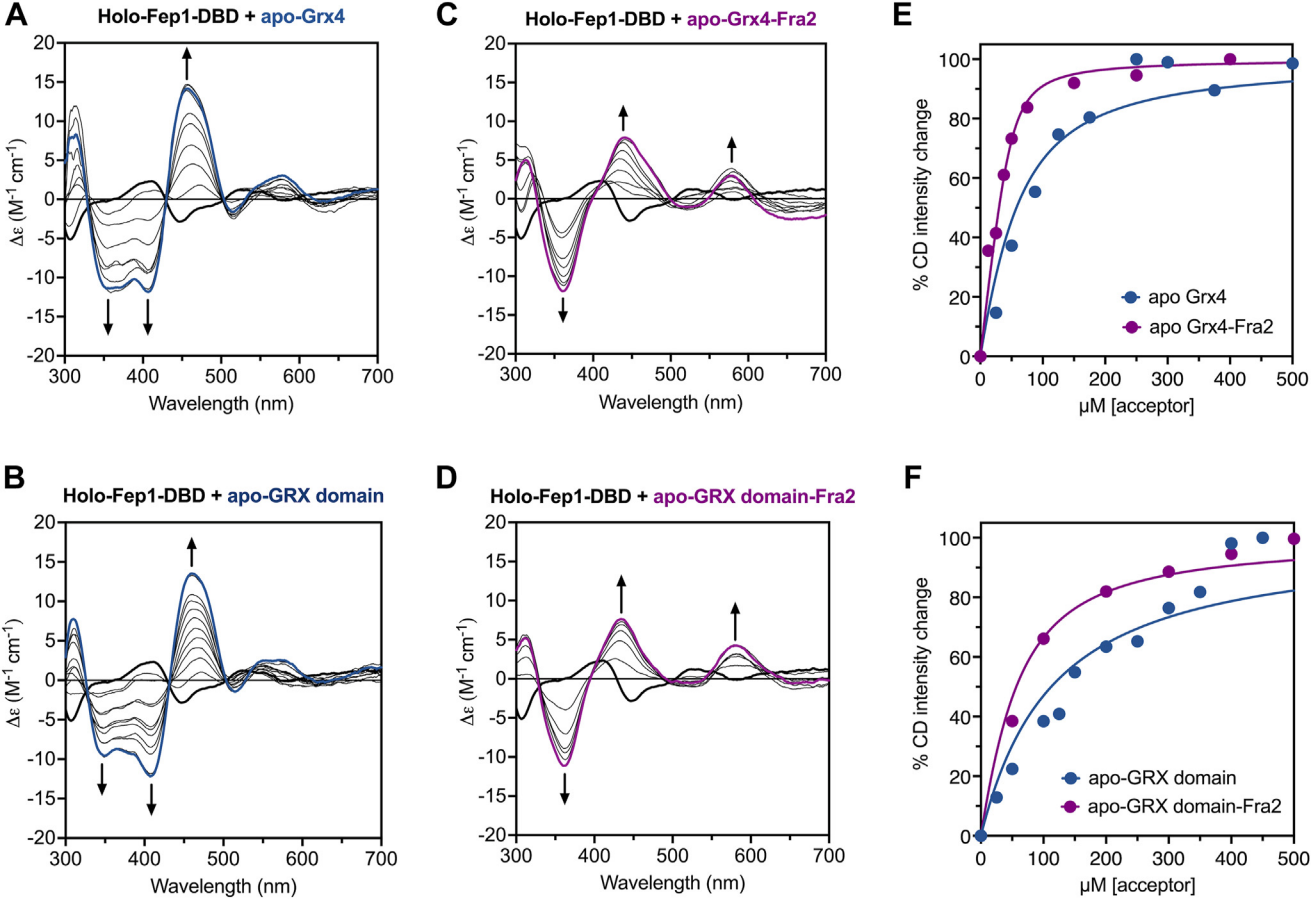
**CD-monitored titration of [2Fe-2S],Zn-Fep1-DBD with apo-Grx4/GRX domain and apo-Fra2**. [2Fe-2S],Zn-Fep1-DBD was titrated with 0.25 to 12 mol equivalents of apo-Grx4 (*A*), apo-GRX domain (*B*), apo-Grx4-Fra2 (*C*), or apo-GRX domain-Fra2 (*D*). The arrows at selected wavelengths indicate the direction of CD intensity change with increasing apo-acceptor protein. Δε values are based on the [2Fe-2S] cluster concentration (50 μM). In *A–D*, *thick black lines* are the CD spectra of [2Fe-2S],Zn-Fep1-DBD alone, *blue lines* are the final titration mixtures with Grx4 or GRX domain, and *purple lines* are the final titration mixtures with Grx4-Fra2 or GRX domain-Fra2. *E* and *F*, % CD intensity changes (between 408 and 458 nm for *A* and *B*, and between 362 and 436 nm for *C* and *D*) from spectra in *A* and *C* (apo-Grx4 ± apo-Fra2) and *B* and *D* (apo-GRX domain ± apo-Fra2) are plotted as a function of the concentration of apo acceptor (dimer). The points were fit to Equation 8 to calculate the *K*_eq_ for each Fe-S exchange reaction (Table 1).

Since both *in vivo* and *in vitro* studies have established that Grx4 interacts with Fep1 (12, 14), we sought to determine whether this interaction involves Fe-S cluster exchange. We titrated apo-Grx4 into [2Fe-2S], Zn-Fep1-DBD and monitored Fe-S coordination changes *via* CD spectroscopy. Increasing amounts of apo-Grx4 caused a transition from the [2Fe-2S],Zn-Fep1-DBD spectrum to a spectrum that resembles [2Fe-2S]-Grx4 homodimer (compare the blue line in Fig. 6*A* to the blue line in Fig. 1*A*). These CD changes occurred within 10 min of Grx4 addition, indicating rapid Fe-S cluster exchange. Addition of apo-GRX domain to holo-Fep1 generated similar spectral transitions (Fig. 6*B*), suggesting interaction of Fep1 with the acceptor proteins and transfer of the [2Fe-2S] cluster to Grx4 or GRX domain.

Since regulation of Fep1 requires both Grx4 and Fra2 and the three proteins form a heteroprotein complex *in vivo* (13), we next tested how the presence of Fra2 impacted cluster transfer from [2Fe-2S],Zn-Fep1-DBD to apo-Grx4 or apo-GRX domain. We performed CD-monitored titrations of [2Fe-2S],Zn-Fep1-DBD with increasing mole equivalents of apo-Grx4:Fra2 or apo-GRX domain:Fra2 added as 1:1 mixtures (Fig. 6, *C* and *D*). The CD spectra changes clearly demonstrate Fe-S cluster transfer from Fep1 to form [2Fe-2S]-bound Grx4-Fra2 or GRX domain-Fra2 (compare the purple lines in Fig. 6, *C* and *D* to the purple lines in Fig. 1, *A* and *B*). In contrast, addition of apo-Fra2 to holo-Fep1 in the absence of Grx4 or GRX domain generated no significant changes in the CD spectrum (Fig. S5*A*), indicating that Fra2 alone does not bind to or perturb the [2Fe-2S] cluster in Fep1. Taken together, these results demonstrate that both full-length Grx4 and GRX domain interact with and receive a cluster from holo-Fep1, in the absence or presence of Fra2, and that Grx4 mediates the Fe-S cluster dependent interaction between Fep1 and Fra2 required for inhibition of Fep1 activity.

The titration plots (Fig. 6, *E* and *F*) and *K*_ex_ values (Table 1) calculated from the Fep1-Grx4-Fra2 titration experiments in Figure 6 provide insight into the energetics of the Fe-S exchange reactions. The *K*_ex_ for holo-Fep1 titrated with full-length Grx4 in the absence of Fra2 (5.3) is 3.8 times greater than the truncated GRX domain (1.4), indicating that Fe-S cluster transfer to full-length Grx4 is more thermodynamically favored. Moreover, addition of Fra2 with Grx4 or GRX domain further enhances the *K*_ex_ of Fe-S cluster exchange with Fep1-DBD by 7-fold (36.1) compared to Grx4 alone, or 2.8-fold (3.9) compared to GRX domain alone (Table 1). Overall, these measurements suggest that both the GRX and TRX domains of Grx4 have a role in recognizing and facilitating Fe-S cluster transfer from Fep1 since transfer is more thermodynamically favorable with the full length protein. Furthermore, the presence of Fra2 significantly enhances transfer of the Fe-S cluster from Fep1, supporting the essential role of Fra2 in regulating Fep1 activity.

### Fe-S cluster transfer between Fep1 and Grx4-Fra2 is unidirectional and requires GSH

Previous researchers hypothesized a possible bidirectional Fe-S cluster transfer between Fep1 and Grx4:Fra2, in which, under iron excess, a cluster may be donated to Fep1 by Grx4-Fra2 to activate its repressor activity (17). If this occurs, it would be similar to the regulation mechanism in *S. cerevisiae*, in which Grx3/4 and Fra2 transfer [2Fe-2S] clusters to the transcriptional activators Aft1/Aft2 under iron replete conditions (7, 29). Therefore, we also tested whether [2Fe-2S]-Grx4/GRX domain or [2Fe-2S]-Grx4/GRX domain-Fra2 can transfer Fe-S clusters to Zn-Fep1. However, we observed no change in CD signals in these experiments with increasing Zn-Fep1 (Fig. S5, *B*–*E*), indicating that cluster transfer between Fep1 and Grx4-Fra2 is unidirectional from Fep1 to Grx4-Fra2 under the conditions tested.

We next tested the role of GSH in Fe-S cluster transfer between Fep1 and Grx4-Fra2. In [2Fe-2S]-bridged Grx-BolA heterocomplexes, GSH serves as one of the Fe-S cluster ligands along with the CGFS motif in the Grx partner and two ligands from the BolA partner (6, 8, 18, 19, 21, 30). To confirm that GSH is required for Fe-S cluster formation on Grx4 or Grx4-Fra2 following transfer from Fep1-DBD, we performed CD-monitored titrations with increasing amounts of apo Grx4/GRX domain or apo Grx4/GRX domain:Fra2 in the absence of GSH (Fig. S6). We observed that Fe-S cluster transfer did not occur under these conditions, confirming that GSH is required as a ligand to form the [2Fe-2S]-Grx4/GRX domain or [2Fe-2S]-Grx4/GRX domain-Fra2 complexes.

## Discussion

The multidomain monothiol glutaredoxin Grx4 and BolA-like protein Fra2 of *S. pombe* function together in regulating iron levels in iron-deplete cells by interacting with and inactivating the transcriptional repressor Fep1 (13, 14). This inactivation mechanism is proposed to involve transfer of either iron or an Fe-S cluster from Fep1 to Grx4-Fra2 under iron-depleted conditions (2, 12, 13, 17); however, this hypothesis has not been systematically tested. To resolve this issue, we aimed to characterize the molecular details of the physical and functional interaction between Grx4, Fra2, and Fep1 to better understand the mechanism for sensing and regulating intracellular iron levels in fission yeast. Our previous biochemical analysis of *S. pombe* Grx4 demonstrated that this Fe-S cluster chaperone forms a [2Fe-2S]-bridged homodimer with two Cys residues from the CGFS motif of Grx4 and two GSH molecules serving as the cluster ligands (11). In *S. cerevisiae*, we have shown that Grx3 and Grx4 also form [2Fe-2S]-bridged heterodimers with Bol2 (previously known as Fra2). In this case, Bol2 replaces a Grx3/4 monomer and one GSH, providing ligation of the cluster *via* His103 and possibly Cys66 (4, 6). Formation of this heterodimeric complex is required for the iron-dependent inhibition of Aft1/Aft2 activity to deactivate iron uptake (4). Here, we used site-directed mutagenesis coupled with biochemical and spectroscopic characterization to confirm that Cys29 and His66 in Fra2 are Fe-S cluster ligands and aid in formation of the Grx4-Fra2 complex in *S. pombe.* Interestingly, our results demonstrate that substitution of either His66 or Cys29 in Fra2 does not prohibit [2Fe-2S] cluster binding between Fra2 and Grx4 (similar to results reported for the *S. cerevisiae* orthologs), but does weaken the interaction between these binding partners. Accordingly, the CD spectrum of [2Fe-2S]-Grx4-Fra2 is significantly different from the spectra of [2Fe-2S]-Grx4-Fra2(H66A) and [2Fe-2S]-Grx4-Fra2(C29A), suggesting differences in the cluster coordination environment for each complex. In contrast, the spectrum of [2Fe-2S]-Grx4 is not altered by titration with Fra2(C29A,H66A), indicating that substitution of both residues abolishes the Grx4-Fra2 interaction, as we observed with the *S. cerevisiae* orthologs (4). WT Fra2 binds with 1:1 stoichiometry to the GRX domain under the CD titration conditions, whereas higher concentrations of Fra2(C29A) or Fra2(H66A) are required to achieve completion of the Fe-S cluster coordination changes. Furthermore, our *K*_*ex*_ measurements suggest that the GRX domain-Fra2(H66A) [2Fe-2S]-bound complex is less stable than the GRX domain-Fra2(C29A) complex, since higher Fra2 concentrations were required to reach saturation for the latter. This observation suggests that the His66 residue plays a more important role in stabilizing the heterocomplex, whereas Cys29 may be weakly coordinated or easily exchangeable.

Analysis of Fra2 function *in vivo* also revealed the critical role of these Fe-S ligands, supporting the results of our biochemical and spectroscopic analysis. Strains expressing the Fra2 C29A, H66A, and C29A/H66A variants grew poorly in the presence of the Fe chelator Dip, unlike strains with WT Fra2. This result suggests that these Fra2 mutants are unable to inactivate Fep1 repressor activity, leading to constitutive repression of high affinity iron uptake genes, even during iron starvation. The mRNA analysis of Fep1-regulated *fio1*^*+*^ and *frp1*^*+*^ gene expression (encoding for iron uptake components) confirmed this hypothesis since these genes remained repressed even in the presence of Dip with Fra2(H66A), Fra2(C29A), or Fra2(H66A,C29A) expression. A similar defect in derepression of Fep1-regulated genes was reported for *S. pombe* strains expressing Grx4(C172S) that lacks the Fe-S ligating residue in the GRX domain (12, 14). Taken together, these results strongly suggest that stable [2Fe-2S] cluster binding by the Fra2-Grx4 complex is required to deactivate the repressor function of Fep1.

One subtle difference we noted between the role of Fra2/Bol2 in iron regulation in *S. pombe versus S. cerevisiae* is the impact of the conserved Cys residue. In *S. cerevisiae* cells, substitution of the conserved Cys66 had no observable effect on the ability of Bol2 to deactivate the transcription factor Aft1 in response to iron sufficiency, whereas substitution of the conserved His103 abolished this activity (4). Based on these results, we concluded that this Cys was not essential for [2Fe-2S] cluster binding by Bol2, which is required for regulation of Aft1 activity. In contrast, expression of the analogous Fra2(C29A) mutant in *S. pombe* impaired depression of Fep1-regulated genes under iron deficiency, suggesting that this residue was important for Fra2 function in *S. pombe*. However, we note that this effect was markedly weaker than the impact of the Fra2(H66A) and Fra2(H66A,C29A) mutants on Fep1 function (Fig. 5) suggesting that His66 was more important for Fra2 function that Cys29. These differences between the *S. pombe* Fra2 variants correlates with the stronger [2Fe-2S] binding thermodynamics of the C29A variant relative to the other variants. In any case, these observations support the finding that the conserved His ligand in Fra2/Bol2 is more critical for stabilizing the [2Fe-2S]-bridged Grx4-Fra2 complex than the conserved Cys.

Our analysis of the interaction between Fep1 and Grx4-Fra2 further illuminates the molecular roles of Grx4 and Fra2 in regulating Fep1 activity in response to iron bioavailability. Previous *in vivo* studies established that both Grx4 and Fra2 physically interact with Fep1 and are essential for inactivation of Fep1 under iron sufficiency (12, 13, 14); however, the nature of this interaction was unclear. In particular, the specific Fe cofactor bound by *S. pombe* Fep1 was somewhat controversial since one group reported that reconstituted Fep1 binds a mixture of [2Fe-2S], [3Fe-4S], and [4Fe-4S] clusters (17), while another group reported that Fep1 binds mononuclear iron (12). Our biochemical and spectroscopic analyses suggest that the interaction between Fep1 and Grx4-Fra2 specifically involves unidirectional [2Fe-2S] cluster exchange from Fep1 to Grx4 or Grx4-Fra2. The Fe-S cluster exchange reaction is thermodynamically most favorable with full-length Grx4 in the presence of Fra2 and GSH, facilitating formation of the GSH-ligated, Grx4-Fra2 heterodimer. The requirement for both the N-terminal TRX domain and the C-terminal GRX domain in Grx4 for efficient transfer is consistent with previous yeast two-hybrid and coimmunoprecipitation assays demonstrating that both Grx4 domains are required for maximal binding to Fep1 (14). Furthermore, the greater thermodynamic stability of the Grx4-Fra2 heterodimer as a cluster acceptor compared to the Grx4 homodimer parallels findings we and others have reported when comparing the stabilities of homologous CGFS Grx homodimers and Grx-BolA heterocomplexes from other organisms (4, 6, 18, 20, 22). In the absence of structural information on Fep1 and Grx4-Fra2, the specific protein-protein interactions and/or conformational changes that drive Fe-S cluster transfer between these proteins is unclear. However, it is likely this involves specific recognition that facilitates a ligand exchange reaction between Fep1 and Grx4-Fra2 to move the cluster between these interaction partners.

**Figure 7:**
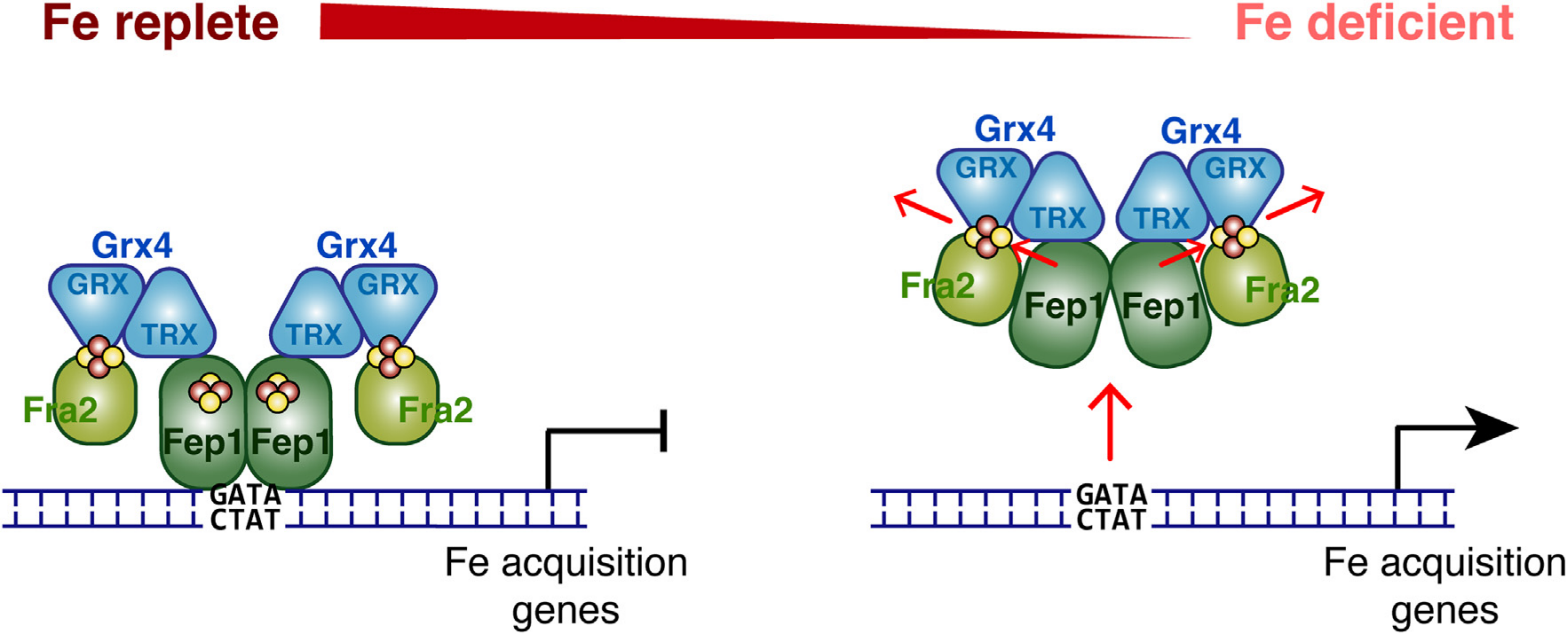
**Model for Grx4-Fra2 dependent regulation of Fep1 repressor activity**. See Discussion for details.

Overall, these findings refine the current mechanistic model for regulation of Fep1 activity by Grx4-Fra2 in response to iron (Fig. 7). Under iron replete conditions when the intracellular Fe-S cluster bioavailability is high, the Grx4-Fra2 complex and Fep1 are each proposed to harbor [2Fe-2S] clusters. [2Fe-2S] binding to Fep1 is proposed to induce a conformation that promotes its binding to the promoters of iron acquisition genes and/or its interaction with transcriptional corepressors (1, 2, 8, 12, 13, 14, 15). *In vivo* evidence suggests that the TRX domain of Grx4 remains bound to Fep1 under these conditions, while binding of the [2Fe-2S] cluster to Grx4-Fra2 may preclude interaction between Fep1 and the GRX domain (14). The observation that Fep1-regulated genes are constitutively repressed in *grx4Δ* and *fra2Δ* strains since Fep1 remains DNA-bound (12, 13, 14) suggests that Fra2 and Grx4 do not play a role in assembling or delivering the Fe-S cluster to Fep1 during iron sufficiency. The Fe-S cluster transfer assays reported here support these *in vivo* findings since Grx4 and Fra2 are unable to efficiently transfer Fe-S clusters to Fep1. Therefore, the specific Fe-S cluster trafficking pathway that metalates Fep1 remains unclear. In any case, when iron levels drop, Fe-S cluster bioavailability is likely limited as Fe-S cluster assembly slows down. Under these conditions when Fe-S cluster occupancy of the Grx4-Fra2 complex decreases, apo Grx4-Fra2 catalyzes removal of the Fe-S cluster from Fep1 to deactivate this repressor. This step proceeds rapidly and is likely under thermodynamic control, as suggested by our *K*_ex_ measurements. The thermodynamics of this reaction may be further favored by the protein abundances of Fep1 and Grx4-Fra2 *in vivo*, since Fep1 is found at relatively low levels (2800 molecules/cell) compared to Grx4 (18,700 molecules/cell) and Fra2 (15,800 molecules/cell) (31). We propose that this Fe-S cluster exchange reaction occurs *via* associative ligand exchange as demonstrated for other metal and metallocofactor trafficking pathways (32, 33, 34, 35) to protect the Fe-S cluster from release to the nuclear/cytosolic compartment. In the future, structural characterization of Fep1 and its binding interface with Grx4-Fra2 is required to uncover the specific protein-protein interactions and/or conformational changes that drive this exchange reaction.

## Experimental procedures

### Bacterial expression plasmid construction

*Escherichia coli* expression plasmids pRSFDuet1-6xHis-Grx4 and pRSFDuet1-6xHis-Fra2 were constructed by inserting the *S. pombe grx4*^*+*^ coding sequence amplified from genomic DNA and the *fra2*^*+*^ coding sequence amplified from a *S. pombe* complementary DNA (cDNA) library (American Type Culture Collection #87284), respectively. Once isolated and purified, the two coding sequences were inserted into the BamHI and SalI sites of pRSFDuet1, generating Grx4 or Fra2 with an N-terminal hexahistidine tag. Fra2 variants H66A and C29A were created by site-directed mutagenesis (QuikChange II Mutagenesis Kit, Agilent) of pRSFDuet1-6xHis-Fra2 using primers listed in Table S1. The Fra2 C29A,H66A double mutant was created by mutagenesis with the C29A primers using pRSFDuet1-6xHis-Fra2(H66A) as the template DNA. To generate pRSFDuet1-His6x-Grx4(Δ1-142) expressing the C-terminal GRX domain of Grx4, a BamHI site was inserted *via* site-directed mutagenesis after the TRX domain sequence using pRSFDuet1-His6x-Grx4 as the template. The resulting plasmid was digested with BamHI and religated to remove the TRX domain located between the two BamHI sites. The pET21b-Fep1-6xHis and pRSFDuet1-6xHis-Fep1-DBD plasmids expressing full length Fep1 with a C-terminal hexahistidine tag or the N-terminal DNA binding domain (2–241) of Fep1 with an N-terminal hexahistidine tag were constructed by inserting the respective *S. pombe fep1*^*+*^ coding sequences amplified from genomic DNA into the NheI and XhoI sites of pET21b or the BamHI and PstI sites of pETDuet1, respectively. All plasmid constructs were confirmed by restriction enzyme digest and Sanger sequencing (GENEWIZ). A list of all plasmids used in this study is shown in Table S2.

### Recombinant protein expression and purification

His-tagged full-length Grx4 and the truncated GRX domain were expressed by transforming pRSFDuet1-6xHis-Grx4 or pRSFDuet1-6xHis-Grx4 (Δ1–142) in *E. coli* strain PK11466, a derivative of BL21(DE3). This strain is engineered to have enhanced expression of the Suf Fe-S biogenesis machinery to generate Fe-S cluster-containing proteins more efficiently (36). Freshly transformed cells were used to inoculate 1 L of LB medium with 30 μg/ml of kanamycin, 1 mM ferric citrate, and 1 mM l-cysteine in a 3-L baffled flask. The cells were grown with shaking (200 rpm) at 37 °C and induced with 1 mM IPTG at *A*_600_ ∼0.7 to 0.8. Post induction, the cultures were grown overnight at 25 °C with shaking (150 rpm) and harvested by centrifugation. Subsequent steps were performed in the glove box (O_2_ < 5 ppm) (Coy Laboratory Products, Inc) and in airtight septum bottles. The pelleted cells were resuspended in 50 ml of Buffer A [50 mM Tris-Mes, pH 7.9, 300 mM NaCl, 20 mM imidazole, and 5% glycerol] with 5 mM GSH and 30 μg/ml PMSF, followed by intermittent sonication and centrifugation to remove cell debris. The cell-free extract was loaded onto a 20-ml HisPrep FF 16/10 column (Cytiva) equilibrated with Buffer A with 5 mM GSH. The protein was eluted with a 20 to 500 mM imidazole gradient and the purest fractions of Grx4 with reddish-brown color, as judged by SDS-PAGE, were pooled together, and concentrated to 1 ml followed by buffer exchange with Buffer B [50 mM Tris-Mes, pH 7.9, 150 mM NaCl, and 5% glycerol] using 10 kDa or 3 kDa molecular weight cutoff (MWCO) centrifugal filters (Millipore Amicon Ultra) to remove imidazole and stored at −80 °C. Grx4 and GRX domain purified this way typically bound 0.3 to 0.4 [2Fe-2S] cluster per dimer. For purifying apo-Grx4/GRX domain, the procedure is similar to holo-Grx4/GRX domain, except the purification was performed aerobically.

Fra2 and its variants were overexpressed by transforming pRSFDuet1-6xHis-Fra2 and the appropriate plasmids for Fra2 variants into the *E. coli* strain BL21(DE3). Freshly transformed cells were used to inoculate 1 L of LB medium with 30 μg/ml kanamycin and grown with shaking (200 rpm) at 37 °C. The cells were induced with 1 mM IPTG at *A*_600_ ∼0.6 to 0.8 and grown overnight at 30 °C with shaking (150 rpm) and harvested by centrifugation. The pelleted cells were resuspended in 50 ml of Buffer A with 2 mM tris(2-carboxyethyl)phosphine (TCEP) and 30 μg/ml PMSF, followed by intermittent sonication and centrifugation to remove cell debris. The cell-free extract was loaded onto a 20-ml HisPrep FF 16/10 column equilibrated with Buffer A with 5 mM TCEP. The protein was eluted with a 20 to 500 mM imidazole gradient and the purest fractions of Fra2 and its variants as judged by SDS-PAGE, were pooled together, and concentrated to 1 ml followed by buffer exchange with Buffer B in 3 kDa MWCO centrifugal filters (Millipore Amicon Ultra) to remove imidazole and TCEP and stored at −80 °C.

Fep1-6xHis and 6xHis-Fep1-DBD were overexpressed by transforming pET21b-Fep1-6xHis or pRSFDuet1-6xHis-Fep1-DBD into *E. coli* strain PK11466. Freshly transformed cells were used to inoculate 1 L of LB medium with 50 μg/ml ampicillin or 30 μg/ml kanamycin, respectively, 1 mM ferric citrate, 1 mM L-cysteine and 100 μM ZnCl_2_ in a 3-L baffled flask. The cells were grown with shaking (200 rpm) at 37 °C and induced with 1 mM IPTG at *A*_600_ ∼ 0.7 to 0.8. Post induction, the cultures were grown overnight at 30 °C with shaking (150 rpm) and harvested by centrifugation. Subsequent steps were performed in the anaerobic glove box to obtain Fe-S bound Fep1/Fep1-DBD. The pelleted cells were resuspended in 50 ml of Buffer C [50 mM Tris-Mes, pH 7.9, 150 mM NaCl, 2 mM GSH], Pierce protease inhibitor tablets (Thermo Fisher Scientific) and 30 μg/ml PMSF, followed by intermittent sonication and centrifugation to remove cell debris. The supernatant was treated with 0.1% streptomycin sulfate for 40 min on ice to remove any DNA contamination followed by centrifugation. The cell-free extract was loaded onto a 5-ml HiTrap Heparin HP column (Cytiva) equilibrated with Buffer C. The protein was eluted with a 150 to 1000 mM NaCl gradient and the purest fractions of Fep1 and Fep1-DBD with reddish-brown color, as judged by SDS-PAGE, were pooled together and concentrated to 1 ml in Buffer C with 5% glycerol using 30 kDa and 10 kDa MWCO centrifugal filters, respectively, and stored at −80 °C. SDS-PAGE analysis of the purified proteins used in this study are shown in Fig. S1.

Purification of Zn-Fep1-DBD was identical to the procedures described for [2Fe-2S],Zn Fep1-DBD with the following exceptions: ferric citrate and L-cysteine were omitted from the growth medium, and pelleted cells were resuspended in 50 ml of Buffer C with 2 mM TCEP rather than GSH. All purification steps were performed aerobically rather than anaerobically. The cell-free extract was loaded onto a 5-ml HiTrap Heparin HP Column equilibrated with Buffer C with 2 mM TCEP. The purified protein was colorless with ∼0.02 Fe/protein and ∼0.6 zinc/protein.

### Analytical and spectroscopic methods

We calculated the concentration of proteins using the theoretical extinction coefficient for the specific apo-proteins. Proteins were diluted in 50 mM Tris-Mes, pH 7.9, 150 mM NaCl, and 6 M guanidine-HCl and the absorbance measured at 280 nm. This value was used to standardize the Bradford assay (Bio-Rad). We found that the Bradford assay underestimates the Grx4 protein concentration by a factor of 1.9, Fra2 by a factor of 1.72, GRX domain by a factor of 1.08, and Fep1-DBD by 1.2 when using bovine serum albumin as the protein standard. Iron concentrations were determined using the colorimetric ferrozine assay (37, 38). Alternatively, inductively coupled plasma MS was performed to confirm the iron and zinc content in purified proteins (Mass Spectrometry Center, University of South Carolina). Inorganic sulfur was detected in protein samples using an acid-labile sulfide assay (39). CD and UV–visible absorption spectra were recorded at room temperature under anaerobic conditions using a Jasco J-815 spectropolarimeter (JASCO) and a Shimadzu UV 1800 spectrophotometer, respectively. Analytical gel filtration was carried out using a Superdex 75 10/300 GL column at a flow rate of 0.5 ml/min in 50 mm Tris-Mes, pH 7.9, 300 mM NaCl, and 5 mM GSH, using degassed buffers. Sigma-Aldrich’s Gel Filtration marker kit, which includes cytochrome c from horse heart (12.4 kDa), carbonic anhydrase from bovine erythrocytes (29 kDa), bovine serum albumin (66 kDa) and aprotinin from bovine lung (6.5 kDa) was used for the molecular mass markers. MS analysis of purified proteins was performed using a Thermo Scientific Orbitrap Velos Pro mass spectrometer in the positive ion mode (Mass Spectrometry Center, University of South Carolina). The samples were analyzed by infusion into the ion source in a mobile phase of 60/40 acetonitrile/water containing 0.1% formic acid.

### CD monitored titration of [2Fe–2S]-Grx4 with Fra2 and its variants

The titrations of [2Fe-2S] Grx4 with Fra2 and its variants were monitored under anaerobic conditions at room temperature using UV-visible CD spectroscopy. The [2Fe-2S]-Grx4 and [2Fe-2S]-GRX domain samples used in the titrations typically contained 0.3 to 0.4 [2Fe-2S] cluster per dimer. Reactions were carried out in a buffer containing 50 mM Tris-Mes, pH 7.9, 300 mM NaCl, and 5 mM GSH, with the [2Fe-2S] cluster concentration kept constant at 50 to 60 μM and the concentration of apo-Fra2 variants varied from 25 to 1250 μM. Following addition of Fra2 variants to [2Fe-2S]-Grx4 in a 1-cm quartz cuvette, samples were mixed thoroughly, capped, and incubated on ice for 10 min before recording the CD spectrum.

### CD-monitored titration of Grx4, Fra2, and Fep1

The titrations of different combinations of apo and holo-Fep1, Grx4, and Fra2 were monitored under anaerobic conditions at room temperature using UV–visible CD spectroscopy. The [2Fe-2S],Zn-Fep1 sample used in the titrations typically contained 0.6 to 0.8 [2Fe-2S] cluster per dimer, whereas the holo-Grx4 had 0.2 to 0.4 [2Fe-2S] cluster per dimer. Reactions were carried out in buffer containing 50 mM Tris–Mes, pH 7.9, 500 mM NaCl, 5 mM GSH, and 5% glycerol, with the [2Fe-2S]^2+^ cluster concentration of the donor protein kept constant at 50 μM and the concentration of apo acceptor proteins varied from 12.5 to 600 μM. Following addition of the Fe-S donor and acceptor proteins into the 1-cm quartz cuvette, the solutions were mixed thoroughly, capped, and incubated on ice for 10 min before recording the CD spectra.

### Measurement of K_ex_ for Fep1-Grx4-Fra2 Fe-S cluster binding interactions

To evaluate the energetics of Fe-S cluster exchange between Fep1, Grx4, and Fra2, we measured the equilibrium constants (*K*_ex_) (40) from the CD-monitored Fe-S cluster exchange experiments described above. The equilibrium expression for the titration of [2Fe-2S]-Grx4 or [2Fe-2S]-GRX domain homodimer with apo-Fra2 to generate [2Fe-2S]-Grx4-Fra2 or [2Fe-2S]-GRX domain-Fra2 heterodimer is:

(1) $$\begin{array}{c} Grx4\left(FeS\right)Grx4\;+\;Fra2\;⇌\;Grx4\;+\;Grx4\left(FeS\right)Fra2\\ K_{ex}\;=\;\frac{{\left[Grx4\right]}_{eq}[Grx4\left(FeS\right){Fra2]}_{eq}}{[Grx4\left(FeS\right){Grx4]}_{eq}{\left[Fra2\right]}_{eq}} \end{array}$$

Similarly, the expression for titration of [2Fe-2S],Zn-Fep1 with apo-Grx4, apo-GRX domain, apo-Grx4-Fra2, or apo-GRX domain-Fra2 is:

(2) $$\begin{array}{c} Fep1\left(FeS\right)\;+\;acceptor\;⇌\;Fep1\;+\;acceptor\left(FeS\right)\\ K_{ex}\;=\;\frac{{\left[Fep1\right]}_{eq}{\left[acceptor\left(FeS\right)\right]}_{eq}}{{\left[Fep1\left(FeS\right)\right]}_{eq}{\left[acceptor\right]}_{eq}} \end{array}$$

where *acceptor* is a homodimer of Grx4 or GRX domain, or a heterodimer of Grx4-Fra2 or GRX domain-Fra2.

Both Equation 1 and Equation 2 can be rewritten as:

(3) $$\begin{array}{c} donor\left(FeS\right)\;+\;acceptor\;⇌\;donor\;+\;acceptor\left(FeS\right)\\ K_{ex}\;=\;\frac{{\left[donor\right]}_{eq}{\left[acceptor\left(FeS\right)\right]}_{eq}}{{\left[donor\left(FeS\right)\right]}_{eq}{\left[acceptor\right]}_{eq}} \end{array}$$

where the Fe-S *donor* is Fe-S bound Grx4 or Fep1, depending on the experiment.

The values for [*donor*]_eq_, [*donor(FeS)*]_eq_, [*acceptor*]_eq_ and [*acceptor(FeS)*]_eq_ were calculated as:

(4) $${\left[donor\right]}_{eq}\;=\;{\left[donor\right]}_{i}\;+\;{\left[donor\left(FeS\right)\right]}_{i}\;\times \;\Delta CD$$

(5) $${{\left[donor\left(FeS\right)\right]}_{eq}\;=\;\left[donor\left(FeS\right)\right]}_{i}\;\times \;\left(1\;-\;\Delta CD\right)$$

(6) $${\left[acceptor\right]}_{eq}={\left[acceptor\right]}_{i}\;-\;{\left[donor\left(FeS\right)\right]}_{i}\;\times \;\Delta CD$$

(7) $${{\left[acceptor\left(FeS\right)\right]}_{eq}\;=\;\left[donor\left(FeS\right)\right]}_{i}\;\times \;\Delta CD$$

where [*donor*]_I_, [*acceptor*]_i_ and [*donor(Fe-S)*]_i_ are the initial concentrations of apo-donor, apo-acceptor, and [2Fe-2S]-donor proteins used in the titration experiments, and ΔCD is the fractional change in CD signal (% CD change/100). Using these expressions, Equation 3 can be rewritten as:

(8) $$\Delta CD\;=\;\frac{\left(K_{ex}\;\times \;A_{i}\;+\;K_{ex}\;\times \;DFe_{i}\;+\;D_{i}\right)\;+\;\sqrt{{\left(K_{ex}\;\times \;A_{i}\;+\;K_{ex}\;\times \;DFe_{i}\;+\;D_{i}\right)}^{2}\;-\;4\;\times \;\left(K_{ex}\;\times \;DFe_{i}-DFe_{i}\right)\left(K_{ex}\;\times \;A_{i}\right)}}{2\left(K_{ex}\;\times \;DFe_{i}\;-\;DFe_{i}\right)}$$

where *D*_i_ is [*donor*]_i_, *A*_*i*_ is [*acceptor*]_i_ and *DFe*_i_ is [*donor*(Fe-S)]_i_. The CD titration curves were fit to this equation to determine the equilibrium constant for each Fe-S cluster exchange experiment.

### Media, yeast strains, and plasmids

*S. pombe* strains were grown on yeast extract plus supplement medium under nonselective growth conditions, as described previously (41). Strains used for gene cassette integration were grown in Edinburgh minimal medium that was supplemented with the antibiotic G418. Alternatively, Edinburgh minimal medium lacking specific amino acids was used for ensuring chromosomal integration events in transformed cells. Liquid cultures were seeded to an *A*_600_ of 0.5 and grown to exponential phase (*A*_600_ of 0.9) in the presence of FeCl_3_ (100 μM). After washing, aliquots of cultures were either treated with 2,2′-Dip (250 μM) or FeCl_3_ (100 μM), or were left untreated for 90 min.

The FY435 (*h*^*+*^ *his7-366 leu1-32 ura4-Δ18 ade6-M210*), AMY36 (*h*^*+*^ *his7-366 leu1-32 ura4-Δ18 ade6-M210 fra2Δ::KAN*^*r*^), and JFJ195 (*h*^*+*^ *his7-366 leu1-32 ura4-Δ18 ade6-M210 fra2*^*+*^*-myc*_*13*_*::KAN*^*r*^) (13) strains were used in this study. In the case of *fra2* mutant strains harboring single (C29A or H66A) and double (C29A/H66A) mutations at the chromosomal locus of *fra2*, we first used the WT *fra2*^*+*^*-Myc*_*13*_ DNA fragment to create different combinations of site-specific mutations by a PCR overlap extension method (42). The resulting DNA fragments containing the *fra2C29A*, *fra2H66A*, and *fra2C29A/H66A* mutant alleles with thirteen copies of the Myc epitope and the *fra2*^*+*^ promoter region were isolated and inserted into the NotI/EcoRI-cut pKSloxP-Kan^r^-loxP plasmid (43), creating plasmids pKSfra2prom-fra2C29A-Myc_13_-loxP-Kan^r^-loxP, pKSfra2prom-fra2H66A-Myc_13_-loxP-Kan^r^-loxP, and pKSfra2prom-fra2C29A/H66A-Myc_13_-loxP-Kan^r^-loxP. Subsequently, a DNA fragment located just downstream of the stop codon of the *fra2*^*+*^ gene was isolated by PCR and then inserted into each of the three above-mentioned plasmids at the SalI and Asp718 sites. These new plasmids were subsequently digested with NotI and Asp718 to produce three DNA fragments that allowed homologous integration of each *fra2* mutant allele (*fra2C29A*, *fra2H66A*, and *fra2C29A/H66A*) at the chromosomal locus of *fra2*^*+*^, thereby replacing its WT coding sequence by the *fra2* mutant alleles containing site-specific mutations (C29A, H66A, and C29A/H66A).

### RNA isolation and analysis

Total RNA was isolated from the indicated cell cultures by extraction using the hot phenol method as described previously (44). Reverse transcription reactions followed by cDNA synthesis were performed as described previously (45). Quantitative PCR reactions that include cDNAs, forward and reverse primers, and a Supermix buffer containing SYBR Green, dNTPs, and a thermostable DNA polymerase were performed using a CFX96 Touch Real-Time PCR System (Bio-Rad) as described previously (45). Results were considered valid if the target-specific fluorescent signal showed a C_t_ value ≤ 37 cycles, and all positive and negative control reactions yielded successful and no amplification, respectively. Fold changes of *frp1*^*+*^ transcript in WT (*fra2*^*+*^) and fra2-C29A, -H66A, and -C29A-H66A mutant samples were calculated using the ΔΔCt method normalized to *act1*^*+*^, the internal control (46, 47, 48). Calculations were performed using the following equation: ΔΔCt = [(Ct gene–Ct ref) in WT] *versus* [(Ct gene–Ct ref) in *fra2Δ* or fra2-C29A, -H66A, and -C29A-H66A mutants) under the indicated experimental conditions that were performed as a function of iron availability. In the case of *frp1*^*+*^, the primer pair allowed the detection of an amplicon corresponding to the coding region between positions +352 and +452 down to the first nucleotide of the initiator codon. To detect the expression of *act1*^*+*^, a primer pair was used for amplifying the coding sequence between +173 and +280 down to the first base of the ATG codon of *act1*^*+*^.

### Western blot analysis

Cell extracts from *S. pombe* strains were prepared with glass beads using a FastPrep-24 instrument (MP Biomedicals). Cells were lysed in TMN_150_ buffer containing 50 mM Tris–HCl (pH 7.5), 150 mM NaCl, 5 mM MgCl_2_, 1% Nonidet P-40, 1 mM PMSF, and a complete protease inhibitor cocktail (P8340, Sigma-Aldrich). Cell lysates were incubated with Triton X-100 (1%) for 30 min on ice prior to being resolved on 10% SDS-polyacrylamide gels. Fra2-Myc_13_ and α-tubulin proteins were detected by immunoblotting with anti-Myc (9E10, Roche Diagnostics) and anti-α-tubulin (B-5-1-2, Sigma-Aldrich) antibodies, respectively.

## Data availability

All data are included in the manuscript.

## Supporting information

This article contains supporting information.

## Conflict of interest

The authors declare there are no conflicts of interest with the contents of this article.

## References

[1] Brault A., Mourer T., Labbé S. (2015). Molecular basis of the regulation of iron homeostasis in fission and filamentous yeasts. IUBMB Life.

[2] Gupta M., Outten C.E. (2020). Iron-sulfur cluster signaling: the common thread in fungal iron regulation. Curr. Opin. Chem. Biol..

[3] Martínez-Pastor M.T., Perea-Garcia A., Puig S. (2017). Mechanisms of iron sensing and regulation in the yeast *Saccharomyces cerevisiae*. World J. Microbiol. Biotechnol..

[4] Li H., Mapolelo D.T., Dingra N.N., Keller G., Riggs-Gelasco P.J., Winge D.R. (2011). Histidine 103 in Fra2 is an iron-sulfur cluster ligand in the [2Fe-2S] Fra2-Grx3 complex and is required for *in vivo* iron signaling in yeast. J. Biol. Chem..

[5] Ueta R., Fujiwara N., Iwai K., Yamaguchi-Iwai Y. (2012). Iron-induced dissociation of the Aft1p transcriptional regulator from target gene promoters is an initial event in iron-dependent gene suppression. Mol. Cell Biol..

[6] Li H., Mapolelo D.T., Dingra N.N., Naik S.G., Lees N.S., Hoffman B.M. (2009). The yeast iron regulatory proteins Grx3/4 and Fra2 form heterodimeric complexes containing a [2Fe-2S] cluster with cysteinyl and histidyl ligation. Biochemistry.

[7] Poor C.B., Wegner S.V., Li H., Dlouhy A.C., Schuermann J.P., Sanishvili R. (2014). Molecular mechanism and structure of the *Saccharomyces cerevisiae* iron regulator Aft2. Proc. Natl. Acad. Sci. U. S. A..

[8] Talib E.A., Outten C.E. (2021). Iron-sulfur cluster biogenesis, trafficking, and signaling: roles for CGFS glutaredoxins and BolA proteins. Biochim. Biophys. Acta Mol. Cell Res..

[9] Mercier A., Watt S., Bahler J., Labbé S. (2008). Key function for the CCAAT-binding factor Php4 to regulate gene expression in response to iron deficiency in fission yeast. Eukaryot. Cell.

[10] Vachon P., Mercier A., Jbel M., Labbé S. (2012). The monothiol glutaredoxin Grx4 exerts an iron-dependent inhibitory effect on Php4 function. Eukaryot. Cell.

[11] Dlouhy A.C., Beaudoin J., Labbé S., Outten C.E. (2017). *Schizosaccharomyces pombe* Grx4 regulates the transcriptional repressor Php4 *via* [2Fe-2S] cluster binding. Metallomics.

[12] Encinar del Dedo J., Gabrielli N., Carmona M., Ayté J., Hidalgo E. (2015). A cascade of iron-containing proteins governs the genetic iron starvation response to promote iron uptake and inhibit iron storage in fission yeast. PLoS Genet..

[13] Jacques J.F., Mercier A., Brault A., Mourer T., Labbé S. (2014). Fra2 is a co-regulator of Fep1 inhibition in response to iron starvation. PLoS One.

[14] Jbel M., Mercier A., Labbé S. (2011). Grx4 monothiol glutaredoxin is required for iron limitation-dependent inhibition of Fep1. Eukaryot. Cell.

[15] Kim K.D., Kim H.J., Lee K.C., Roe J.H. (2011). Multi-domain CGFS-type glutaredoxin Grx4 regulates iron homeostasis *via* direct interaction with a repressor Fep1 in fission yeast. Biochem. Biophys. Res. Commun..

[16] Cutone A., Howes B.D., Miele A.E., Miele R., Giorgi A., Battistoni A. (2016). *Pichia pastoris* Fep1 is a [2Fe-2S] protein with a Zn finger that displays an unusual oxygen-dependent role in cluster binding. Sci. Rep..

[17] Kim H.J., Lee K.L., Kim K.D., Roe J.H. (2016). The iron uptake repressor Fep1 in the fission yeast binds Fe-S cluster through conserved cysteines. Biochem. Biophys. Res. Commun..

[18] Li H., Mapolelo D.T., Randeniya S., Johnson M.K., Outten C.E. (2012). Human glutaredoxin 3 forms [2Fe-2S]-bridged complexes with human BolA2. Biochemistry.

[19] Dlouhy A.C., Li H., Albetel A.N., Zhang B., Mapolelo D.T., Randeniya S. (2016). The *Escherichia coli* BolA protein IbaG forms a histidine-ligated [2Fe-2S]-bridged complex with Grx4. Biochemistry.

[20] Banci L., Camponeschi F., Ciofi-Baffoni S., Muzzioli R. (2015). Elucidating the molecular function of human BOLA2 in GRX3-dependent anamorsin maturation pathway. J. Am. Chem. Soc..

[21] Roret T., Tsan P., Couturier J., Zhang B., Johnson M.K., Rouhier N. (2014). Structural and spectroscopic insights into BolA-glutaredoxin complexes. J. Biol. Chem..

[22] Yeung N., Gold B., Liu N.L., Prathapam R., Sterling H.J., Willams E.R. (2011). The *E. coli* monothiol glutaredoxin GrxD forms homodimeric and heterodimeric FeS cluster containing complexes. Biochemistry.

[23] Trnka D., Hossain M.F., Jordt L.M., Gellert M., Lillig C.H. (2020). Role of GSH and iron-sulfur glutaredoxins in iron metabolism-review. Molecules.

[24] Mühlenhoff U., Braymer J.J., Christ S., Rietzschel N., Uzarska M.A., Weiler B.D. (2020). Glutaredoxins and iron-sulfur protein biogenesis at the interface of redox biology and iron metabolism. Biol. Chem..

[25] Pelletier B., Beaudoin J., Philpott C.C., Labbé S. (2003). Fep1 represses expression of the fission yeast *Schizosaccharomyces pombe* siderophore-iron transport system. Nucleic Acids Res..

[26] Pelletier B., Trott A., Morano K.A., Labbé S. (2005). Functional characterization of the iron-regulatory transcription factor Fep1 from *Schizosaccharomyces pombe*. J. Biol. Chem..

[27] Dailey H.A., Finnegan M.G., Johnson M.K. (1994). Human ferrochelatase is an iron-sulfur protein. Biochemistry.

[28] Gao H., Subramanian S., Couturier J., Naik S.G., Kim S.K., Leustek T. (2013). *Arabidopsis thaliana* Nfu2 accommodates [2Fe-2S] or [4Fe-4S] clusters and is competent for *in vitro* maturation of chloroplast [2Fe-2S] and [4Fe-4S] cluster-containing proteins. Biochemistry.

[29] Li H., Outten C.E. (2019). The conserved CDC motif in the yeast iron regulator Aft2 mediates iron-sulfur cluster exchange and protein-protein interactions with Grx3 and Bol2. J. Biol. Inorg. Chem..

[30] Nasta V., Giachetti A., Ciofi-Baffoni S., Banci L. (2017). Structural insights into the molecular function of human [2Fe-2S] BOLA1-GRX5 and [2Fe-2S] BOLA3-GRX5 complexes. Biochim. Biophys. Acta Gen. Subj..

[31] Marguerat S., Schmidt A., Codlin S., Chen W., Aebersold R., Bahler J. (2012). Quantitative analysis of fission yeast transcriptomes and proteomes in proliferating and quiescent cells. Cell.

[32] Boal A.K., Rosenzweig A.C. (2009). Structural biology of copper trafficking. Chem. Rev..

[33] Osman D., Martini M.A., Foster A.W., Chen J., Scott A.J.P., Morton R.J. (2019). Bacterial sensors define intracellular free energies for correct enzyme metalation. Nat. Chem. Biol..

[34] Camponeschi F., Banci L. (2019). Metal cofactors trafficking and assembly in the cell: a molecular view. Pure Appl. Chem..

[35] Li Z., Mascarenhas R., Twahir U.T., Kallon A., Deb A., Yaw M. (2020). An interprotein Co-S coordination complex in the B(12)-trafficking pathway. J. Am. Chem. Soc..

[36] Corless E.I., Mettert E.L., Kiley P.J., Antony E. (2020). Elevated expression of a functional Suf pathway in *Escherichia coli* BL21(DE3) enhances recombinant production of an iron-sulfur cluster-containing protein. J. Bacteriol..

[37] Fish W.W. (1988). Rapid colorimetric micromethod for the quantitation of complexed iron in biological samples. Methods Enzymol..

[38] Riemer J., Hoepken H.H., Czerwinska H., Robinson S.R., Dringen R. (2004). Colorimetric ferrozine-based assay for the quantitation of iron in cultured cells. Anal. Biochem..

[39] Beinert H. (1983). Semi-micro methods for analysis of labile sulfide and of labile sulfide plus sulfane sulfur in unusually stable iron-sulfur proteins. Anal. Biochem..

[40] Huffman D.L., O'Halloran T.V. (2000). Energetics of copper trafficking between the Atx1 metallochaperone and the intracellular copper transporter, Ccc2. J. Biol. Chem..

[41] Sabatinos S.A., Forsburg S.L. (2010). Molecular genetics of *Schizosaccharomyces pombe*. Methods Enzymol..

[42] Ho S.N., Hunt H.D., Horton R.M., Pullen J.K., Pease L.R. (1989). Site-directed mutagenesis by overlap extension using the polymerase chain reaction. Gene.

[43] Normant V., Beaudoin J., Labbé S. (2015). An antisense RNA-mediated mechanism eliminates a meiosis-specific copper-regulated transcript in mitotic cells. J. Biol. Chem..

[44] Chen D., Toone W.M., Mata J., Lyne R., Burns G., Kivinen K. (2003). Global transcriptional responses of fission yeast to environmental stress. Mol. Biol. Cell.

[45] Brault A., Mbuya B., Labbé S. (2022). Sib1, Sib2, and Sib3 proteins are required for ferrichrome-mediated cross-feeding interaction between *Schizosaccharomyces pombe* and *Saccharomyces cerevisiae*. Front. Microbiol..

[46] Protacio R.U., Mukiza T.O., Davidson M.K., Wahls W.P. (2022). Molecular mechanisms for environmentally induced and evolutionarily rapid redistribution (plasticity) of meiotic recombination. Genetics.

[47] Schmittgen T.D., Livak K.J. (2008). Analyzing real-time PCR data by the comparative C(T) method. Nat. Protoc..

[48] Livak K.J., Schmittgen T.D. (2001). Analysis of relative gene expression data using real-time quantitative PCR and the 2(-Delta Delta C(T)) Method. Methods.

