# Supplementary material for: Iron homeostasis proteins Grx4 and Fra2 control activity of the Schizosaccharomyces pombe iron repressor Fep1 by facilitating [2Fe-2S] cluster removal
Source: J Biol Chem. 2023 Nov 3;299(12):105419. doi: 10.1016/j.jbc.2023.105419 (PMC10704371; doi:10.1016/j.jbc.2023.105419)
Supplement: Supplemental data [file mmc1.pdf]

Supporting Information

Iron homeostasis proteins Grx4 and Fra2 control activity of the *Schizosaccharomyces pombe* iron repressor Fep1 by facilitating [2Fe-2S] cluster removal

Debolina Hati[1], Ariane Brault[2], Malini Gupta[1], Kylie Fletcher[1], Jean-François Jacques[2], Simon Labbé[2], Caryn E. Outten[1]

[1] Department of Chemistry and Biochemistry, University of South Carolina, Columbia, SC 29208, USA

[2] Département de Biochimie et de Génomique Fonctionnelle, Faculté de médecine et des sciences de la santé, Université de Sherbrooke, Sherbrooke, Quebec J1E 4K8, Canada.

Table S1. List of primers used in this study.

Table S2. List of bacterial and yeast expression plasmids used in this study.

Figure S1. SDS-PAGE analysis of purified proteins used in this study.

Figure S2. UV-visible absorption and CD spectra of as-purified *S. pombe* [2Fe-2S]-Grx4 and [2Fe-2S]-GRX domain.

Figure S3. Assessment of the mRNA and protein steady-state levels of Fra2-Myc_{13} and its mutants derivatives.

Figure S4. UV-visible absorption and CD spectra of as-purified *S. pombe* Fep1 and Fep1-DBD (DNA binding domain).

Figure S5. Fe-S cluster exchange between Fep1 and Grx4-Fra2 is unidirectional and requires Grx4.

Figure S6. GSH is required for Fe-S cluster exchange between holo-Fep1-DBD and apo-Grx4/Fra2.

Table S1. List of primers used in this study. Bases altered by site-directed mutagenesis are shown in bold. The BamHI site introduced to remove the TRX domain is underlined.

**Primer Name**	**Primer Sequence**
Primers used for construction of *E. coli* expression plasmids	
Fra2 C29A FOR	TGAGATCCAAGATATGTCTGGAGGA**GC**TGGCCAGAACTTC
Fra2 C29A REV	GAAGTTCTGGCCA**GC**TCCTCCAGACATATCTTGGATCTCA
Fra2 H66A FOR	CTTCAAGAGGTGATCAAGGATATC**GC**TGCCTTTACTCAGAAATGTTAT
Fra2 H66A REV	ATAACATTTCTGAGTAAAGGCA**GC**GATATCCTTGATCACCTCTTGAAG
Grx4 Δ1-142 FOR	TTCCAATACCTCTAAAGCTCCAAATGGGG**GATCC**TTCTGAACTAAATGAAAGGTTGAGCACG
Grx4 Δ1-142 REV	CGTGCTCAACCTTTCATTTAGTTCAGAA**GGATC**CCCCATTTGGAGCTTTAGAGGTATTGGAA
Primers used for PCR overlap extension	
OE fra2 H66A FOR	GTGATCAAGGATATC**GCT**GCCTTTACTCAGAAAT
OE fra2 H66A REV	CATTTCTGAGTAAAGGC**AGC**GATATCCTTGATCAC
OE fra2 C29A FOR	GATATGTCTGGAGGA**GCT**GGCCAGAACTTCGAAG
OE fra2 C29A REV	CTTCGAAGTTCTGGCC**AGC**TCCTCCAGACATATC
OE Fra2 Start CDS FOR	AAGGATCC**ATG**GTAAATGCACAACAACTTGAGCTTTTGATACAAAATACATTAGAACC
OE Fra2 noSTOP CDS REV	TTCCCGGGTTTTGCTTGAAGAGCTTCCCATTGTGCCGG
Primers used for qPCR analysis	
Frp1 qPCR FOR	GCCGCTCGGTTAGGATTT
Frp1 qPCR REV	TCGTGAGACGAAATGAGCAATA
Fra2 qPCR FOR	CCAAGATATGTCTGGAGGATGTG
Fra2 qPCR REV	GATTCACAAGACGGTGACGA
Act1 qPCR FOR	CTCCTGAGCGTAAATACTCTGTC
Act1 qPCR REV	GTCCGCTCTCATCATACTCTT

Table S2. List of bacterial and yeast expression plasmids used in this study.

Plasmid name	Description	Antibiotic resistance
E. coli expression plasmids		
pRSFDuet1-6xHis-Grx4	Expresses full length Grx4 with an N-terminal 6xHis tag	Kan
pRSFDuet1-6xHis-Grx4 (Δ1-142)	Expresses Grx4 C-terminal Grx domain (residues 143-244) with N-terminal 6xHis tag	Kan
pRSFDuet1-6xHis-Fra2	Expresses full length Fra2 with an N-terminal 6xHis tag	Kan
pRSFDuet1-6xHis-Fra2(H66A)	Expresses full length Fra2 with His66 mutated to Ala with an N-terminal 6xHis tag	Kan
pRSFDuet1-6xHis-Fra2(C29A)	Expresses full length Fra2 with Cys29 mutated to Ala with an N-terminal 6xHis tag	Kan
pRSFDuet1-6xHis-Fra2(C29A,H66A)	Expresses full length Fra2 with Cys29 and His66 mutated to Ala with an N-terminal 6xHis tag	Kan
pET21b-Fep1-6xHis	Expresses full length Fep1 with a C-terminal 6xHis tag	Amp
pRSFDuet1-6xHis-Fep1-DBD	Expresses Fep1 DNA binding domain (residues 2-241) with an N-terminal 6xHis tag	Kan
S. pombe expression plasmids		
pKSfra2prom-fra2C29A-Myc_{13}-loxP-Kan^r-loxP-fra2-3'UTR	Produces a DNA fragment that allows homologous integration of fra2C29A allele at the chromosomal locus of *fra2*$^+$	G418
pKSfra2prom-fra2H66A-Myc_{13}-loxP-Kan^r-loxP-fra2-3'UTR	Produces a DNA fragment that allows homologous integration of fra2H66A allele at the chromosomal locus of *fra2*$^+$	G418
pKSfra2prom-fra2C29A/H66A-Myc_{13}-loxP-Kan^r-loxP-fra2-3'UTR	Produces a DNA fragment that allows homologous integration of fra2C29A/H66A allele at the chromosomal locus of *fra2*$^+$	G418

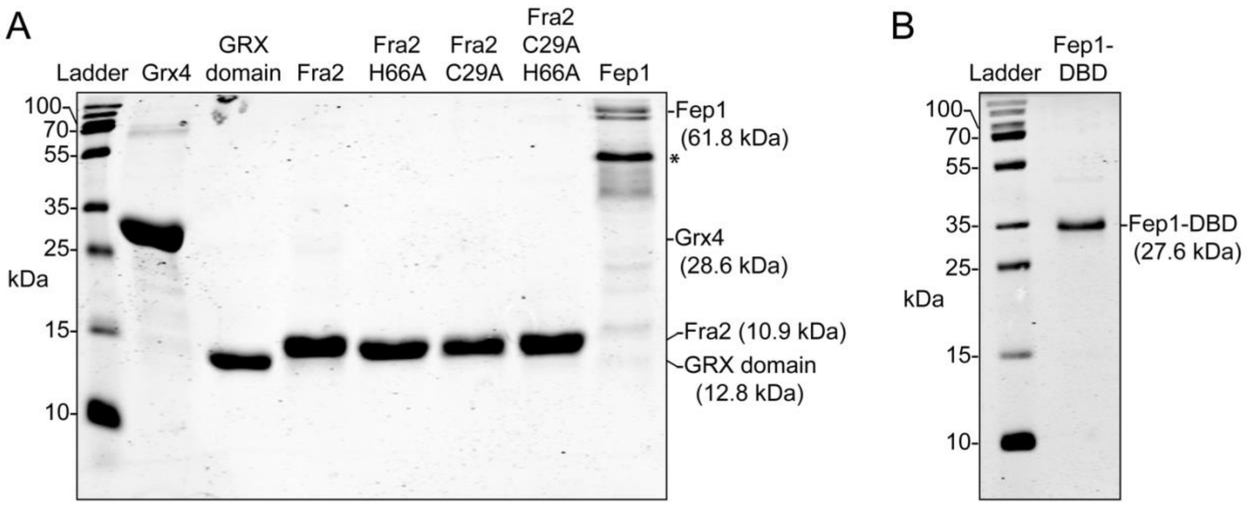

Figure S1. SDS-PAGE analysis of purified proteins used in this study. *A*, As-purified Grx4, GRX domain, Fra2 variants, full-length Fep1, and (*B*) Fep1-DBD were loaded in the indicated lanes. Please note that Fra2 runs higher than expected by SDS-PAGE. In *A*, the band indicated by an asterisk (*) is a proteolysis product of full-length Fep1 verified by MS analysis.

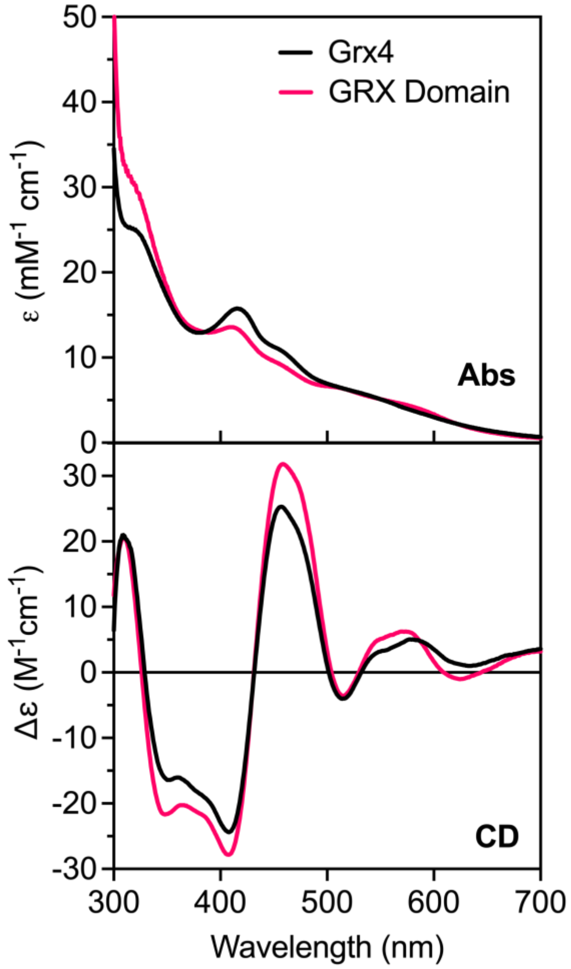

Figure S2. UV-visible absorption (*top*) and CD spectra (*bottom*) of as-purified *S. pombe* [2Fe-2S]-Grx4 (black line) and [2Fe-2S]-GRX domain (dark pink line). ε and Δε values are based on [2Fe-2S] concentration (50 μM).

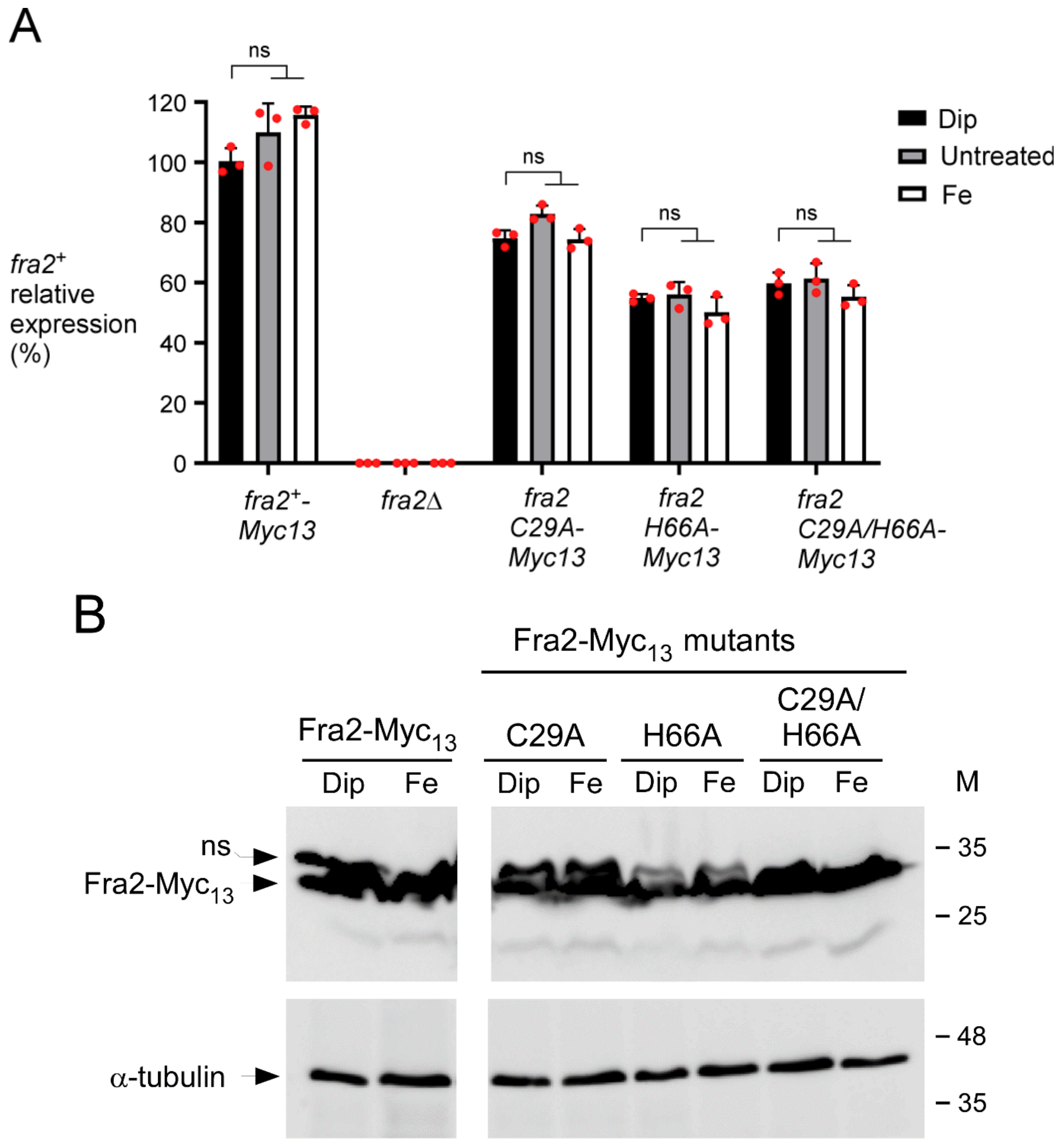

Figure S3. Assessment of the mRNA (*A*) and protein (*B*) steady-state levels of Fra2-Myc_{13} and its mutant derivatives. *A*, Representative expression profile of the *fra2*$^+$ transcript in cells expressing the wild-type *fra2*$^+$-*Myc*$_{13}$ or its mutant derivatives that were left untreated (-) or were incubated in the presence of Dip (250 µM) or $FeCl_3$ (Fe, 100 µM) for 90 min. Total RNA was prepared from culture aliquots, and steady-state mRNA levels of *fra2*$^+$ and *act1*$^+$ were analyzed by RT-qPCR assays. Graphic representation of quantification of three independent RT-qPCR assays. Error bars indicate the standard deviation (± SD; error bars). The mark "ns" stands for not significant (two-way ANOVA with Tukey's multiple comparisons test against the indicated strain grown under low-iron conditions). *B*, Strains expressing the indicated *fra2-Myc*$_{13}$ alleles were treated with Dip or $FeCl_3$ (Fe), as described in panel A. Aliquots of whole cell extract preparations were analyzed by immunoblot assays using anti-Myc and anti-α-tubulin antibodies. The positions of the molecular weight standards (in kDa) are indicated on the right side.

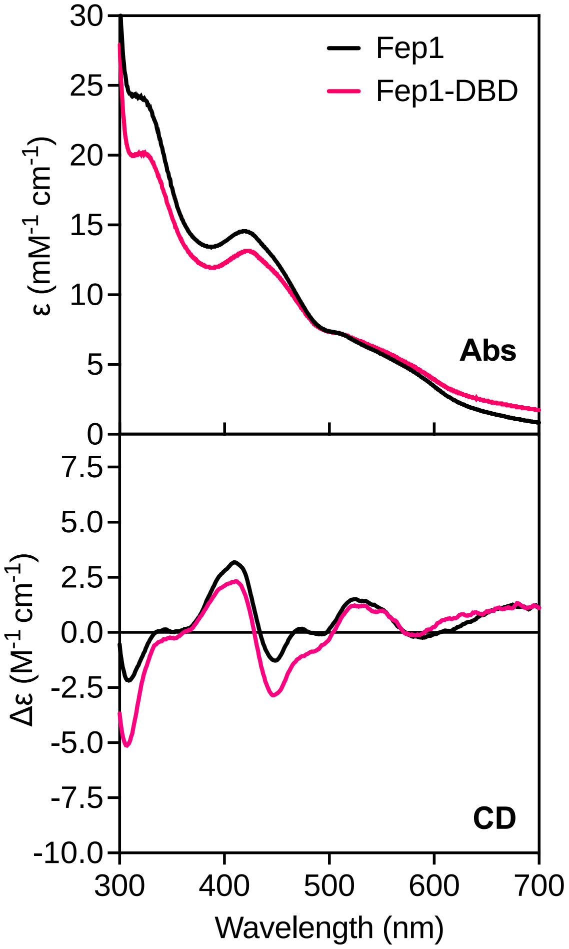

Figure S4. UV-visible absorption (*top*) and CD spectra (*bottom*) of as-purified full length, holo-Fep1 (black line) and holo-Fep1-DBD (dark pink line). ε and Δε values are based on [2Fe-2S] concentration (50 μM).

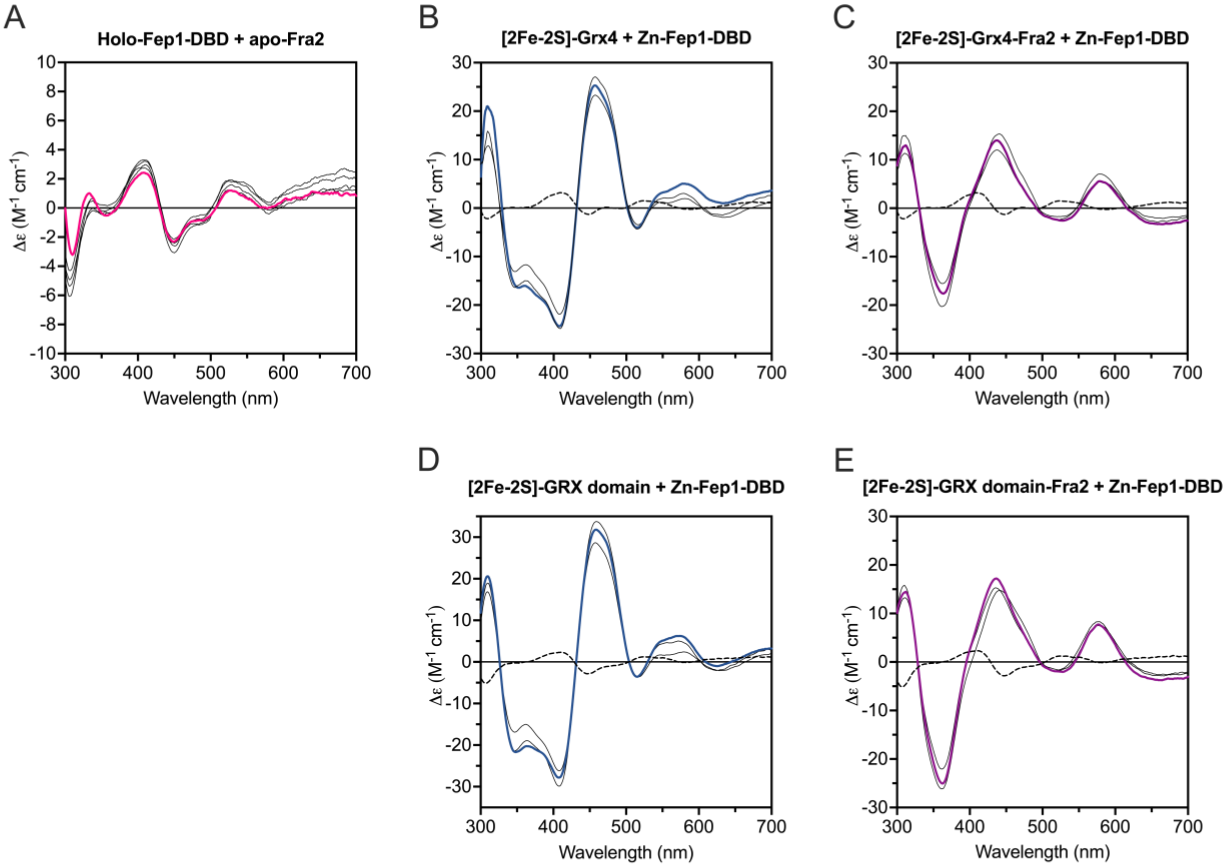

Figure S5. Fe-S cluster exchange between Fep1 and Grx4-Fra2 is unidirectional and requires Grx4. *A*, CD spectra of [2Fe-2S],Zn-Fep1-DBD (pink line) titrated with 1-10 fold excess of apo-Fra2 (thin black lines). CD-monitored titration of [2Fe-2S]-Grx4 (*B*), [2Fe-2S]-Grx4-Fra2 (*C*), [2Fe-2S]-GRX domain (*D*), [2Fe-2S]-GRX domain-Fra2 (*E*), with 1-2 fold excess of Zn-Fep1-DBD (thin black lines). The blue lines in *B*, *D* represent [2Fe-2S]-Grx4 or [2Fe-2S]-GRX domain alone while the purple lines in *C*, *E* represent [2Fe-2S]-Grx4-Fra2 or [2Fe-2S]-GRX domain-Fra2 alone. The dashed lines in *B*-*E* represent the CD spectrum of [2Fe-2S],Zn-Fep1-DBD for reference purposes to depict the spectrum expected if cluster transfer occurred. Δε values are based on the [2Fe-2S] cluster concentration (50 μM).

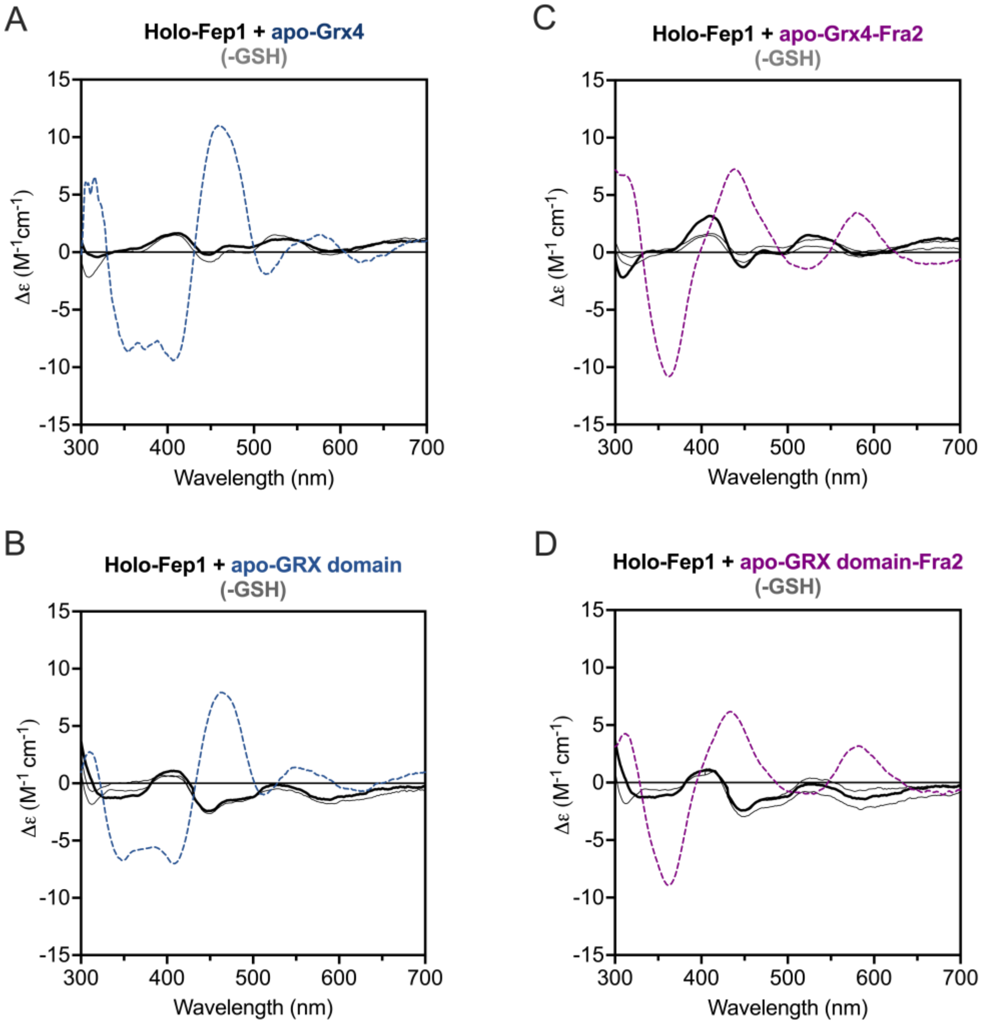

Figure S6. GSH is required for Fe-S cluster exchange between holo-Fep1-DBD and apo-Grx4/Fra2. CD monitored titrations of [2Fe-2S],Zn-Fep1-DBD with apo-Grx4 (*A*), apo-GRX domain (*B*), apo-Grx4-Fra2 (*C*), or apo-GRX domain-Fra2 (*D*) in the absence of GSH. The thick black line in each panel represents [2Fe-2S],Zn Fep1-DBD alone. The thin black lines indicate [2Fe-2S],Zn Fep1-DBD titrated with 0.5-4 mol eq. of apo-Grx4/GRX domain homodimer or apo-Grx4/GRX domain-Fra2 heterodimer. For comparison, the dotted blue lines in *A*,*B* depict the CD spectrum of [2Fe-2S],Zn Fep1-DBD + 3-4 eq. apo-Grx4 or apo-GRX domain homodimer in the presence of GSH (from Fig. 6A,B). Likewise, the dotted purple lines in *C*,*D* represent the CD spectrum of [2Fe-2S],Zn Fep1-DBD + 3-4 eq. apo-Grx4-Fra2 or apo-GRX domain-Fra2 in the presence of GSH (from Fig. 6C,D). Δε values are based on the [2Fe-2S] cluster concentrations (50 μM).
